# Zooplankton biodiversity and temporal dynamics (2005–2015) in a coastal station in western Portugal (Northeastern Atlantic Ocean)

**DOI:** 10.7717/peerj.16387

**Published:** 2023-11-21

**Authors:** Antonina Dos Santos, Raquel Marques, Rita F.T. Pires

**Affiliations:** 1 IPMA, Portuguese Institute for Sea and Atmosphere, Algés, Portugal; 2 CIIMAR—Interdisciplinary Centre of Marine and Environmental Research, Matosinhos, Portugal; 3 Senckenberg am Meer - DZMB, Hamburg, Germany

**Keywords:** Abundance, Biomass, Temporal variability, Cascais Watch, Time series, Lisbon Bay, Trends, Plankton

## Abstract

Long-term monitoring of zooplankton assemblages provides essential knowledge to assess key factors impacting marine ecosystems. Despite the importance of this type of data, monitoring stations worldwide are spatially and temporally limited due to the difficulty of maintaining them. In the northeastern Atlantic area, Cascais-Watch is one monitoring site operating since 2005, despite some constraints throughout the years, and has allowed the collection of important data on the zooplankton communities of the area. The present work summarizes the knowledge collected until 2015 on the biodiversity and dynamics of zooplankton in the site. The results showed a year-round high productivity of the zooplankton abundance, biomass and diversity for the area, with no significant general trends or periodicity, despite the relatively lower winter and higher spring values. The results revealed two main transition periods with marked changes in species composition and dominance of the most abundant taxa. This shift was tentatively attributed to the extended annual dry season verified in Portugal after 2011, the low values of upwelling and precipitation, and the warmer waters. The zooplankton abundance presented an interannual increase for spring periods, and the proportion of Copepoda, the dominant taxa, was lower during summer months, corresponding to increased abundances of Mollusca, Diplostraca (Cladocera) and Cnidaria. In particular, the study shows an increasing abundance of the gelatinous species (particularly Cnidaria) for spring/summer months in recent years, suggesting changes in primary production and prey dynamics. Other relevant tendencies were the higher abundance of meroplankton, such as Bivalvia and fish larvae/eggs, and the decreasing trend in the abundance of the meroplanktonic coastal crustaceans, Decapoda and Cirripedia taxa, highlighting possible changes in the benthic coastal populations in the study region. The present study highlights probable changes and trends in the zooplankton community that should be monitored in the following years.

## Introduction

Coastal areas are complex systems influenced by both marine and land processes, being among the most ecologically and socio-economically vital ecosystems in the world. Given their importance, coastal areas are under major concern regarding the potential impact of climate change and anthropogenic pressures (Harley et al., 2006). Changes in the physical and chemical properties of the marine environment may alter the physiological functioning and behavior of organisms, leading to changes in population dynamics. Consequent modifications in the community structure affect both the bottom-up and top-down processes within the food web (Doney et al., 2012).

Zooplankton play a key role in the coastal pelagic food webs, transferring the energy from primary producers to top predators. Given its quick response to environmental changes, these organisms are good indicators of ecosystem changes (*e.g.*, Muñoz Colmenares, Soria & Vicente, 2021). Therefore, species succession and community shifts may emerge as an outcome of climate change. Long-term changes in zooplankton biomass, diversity and community structure have been frequently associated with climate change and anthropogenic impacts (*e.g.*, Richardson, 2008). These factors may have severe effects on the zooplankton communities (including fish eggs and larvae), potentially reducing food availability or modifying the nutritional quality of these organisms for higher trophic levels (Wright, Pinnegar & Fox, 2020). For these reasons, long-term observations of population and community composition dynamics are regarded as research priorities (Pitois & Yebra, 2022).

Upwelling areas are paramount sources of primary productivity, supporting substantial abundances of zooplankton and prosperous food webs, which ultimately promote favorable conditions for populations of commercially important fish species (Pires & Dos Santos, 2020). The coast of Portugal, in the northern limit of the Northeastern Atlantic Upwelling System (*e.g.*, Relvas et al., 2007), sustains large populations of planktivorous fish such as sardines (*Sardina pilchardus*), anchovies (*Engraulis encrasicolus*) and horse mackerel (*Trachurus trachurus*), with great economic value (Santos et al., 2007). For instance, catches of sardine, the most commercially important and dominant fish species in the area, fluctuates around 150 thousand tonnes per year (Santos et al., 2007). The recruitment of these species is tightly dependent on upwelling (Santos, Borges & Groom, 2001), given the favorable conditions provided for fish larvae in terms of the high food availability (*i.e.,* zooplankton). Nevertheless, despite the acknowledged importance and the works already available (*e.g.*, Lindley & Daykin, 2005; Sobrinho-Gonçalves et al., 2013; Stehle, Dos Santos & Queiroga, 2007; Domínguez et al., 2017; Cruz et al., 2020), studies assessing the seasonal and interannual variability of the composition of zooplanktonic communities off Portugal are still required, given that established monitoring for wider spatial and temporal scales is still not fully implemented in Portuguese waters.

Future climate predictions suggest an intensification of the upwelling events, with potentially severe consequences for planktonic communities (Bakun et al., 2010). The nutrient enrichment effect of stronger upwelling can be counteracted by an increased rate in the offshore transport of organisms (Bakun et al., 2010), such as fish larvae (Santos, Borges & Groom, 2001). Furthermore, under climate change scenarios for Portugal, the decrease in both precipitation (Soares et al., 2015) and the runoff of rivers such as the Tagus have been predicted, with the latter expectedly promoting more saline environmental conditions (Kilsby et al., 2007).

The present study uses data on diversity and temporal variability of zooplankton communities obtained in a highly productive coastal station, Cascais-Watch (hereafter designated as CCW), located in the Cascais Bay (Site 54, O’Brien, Wiebe & Hay, 2021), in the northeastern Atlantic. The station is under the influence of seasonal upwelling, promoted by favorable northerly winds, which are strongest between June and August (Relvas et al., 2007; Pires & Dos Santos, 2020). In addition, it is also influenced by the Tagus estuarine plume that, induced by wind and river runoff, is advected offshore (Vaz et al., 2009; Relvas et al., 2007). Both processes, together with the conspicuous local topographical structures, create complex mesoscale circulation features, shaping the ecosystem and the dynamics of the zooplankton communities. The study station is placed in the northern region of the Lisbon Bay, which was previously suggested as a retention area (*e.g.*, Moita et al., 2003), driven by the sheltering effect of the Estremadura headland (Raso/Roca and Espichel Capes) from the direct influence of the offshore circulation features.

This study represents the first long term comprehensive research of the zooplankton community on the Portuguese coast. Our purpose is to describe the dynamics and composition of the zooplankton community at the CCW station from 2005 to 2015, and assess its temporal (monthly, seasonal and interannual) differences. Considering the available environment variables, we analyzed and discussed their effect on the abundance and taxa composition of the local zooplankton community. The CCW is in a temperate zone and thus our hypothesis is that we will notice a difference between spring/summer and winter periods at least in terms of zooplankton abundance and composition. Since the CCW station is under the influence of coastal upwelling, we also expect to have high productivity during these periods. On the other hand, as the CCW station is placed outside the Tagus estuary, whose discharge flow is dependent on the precipitation regimes, and considering the effects of climate changes, we expect to detect signs of shifts on the taxa composition and abundance through the years.

## Materials & Methods

**Figure 1:**
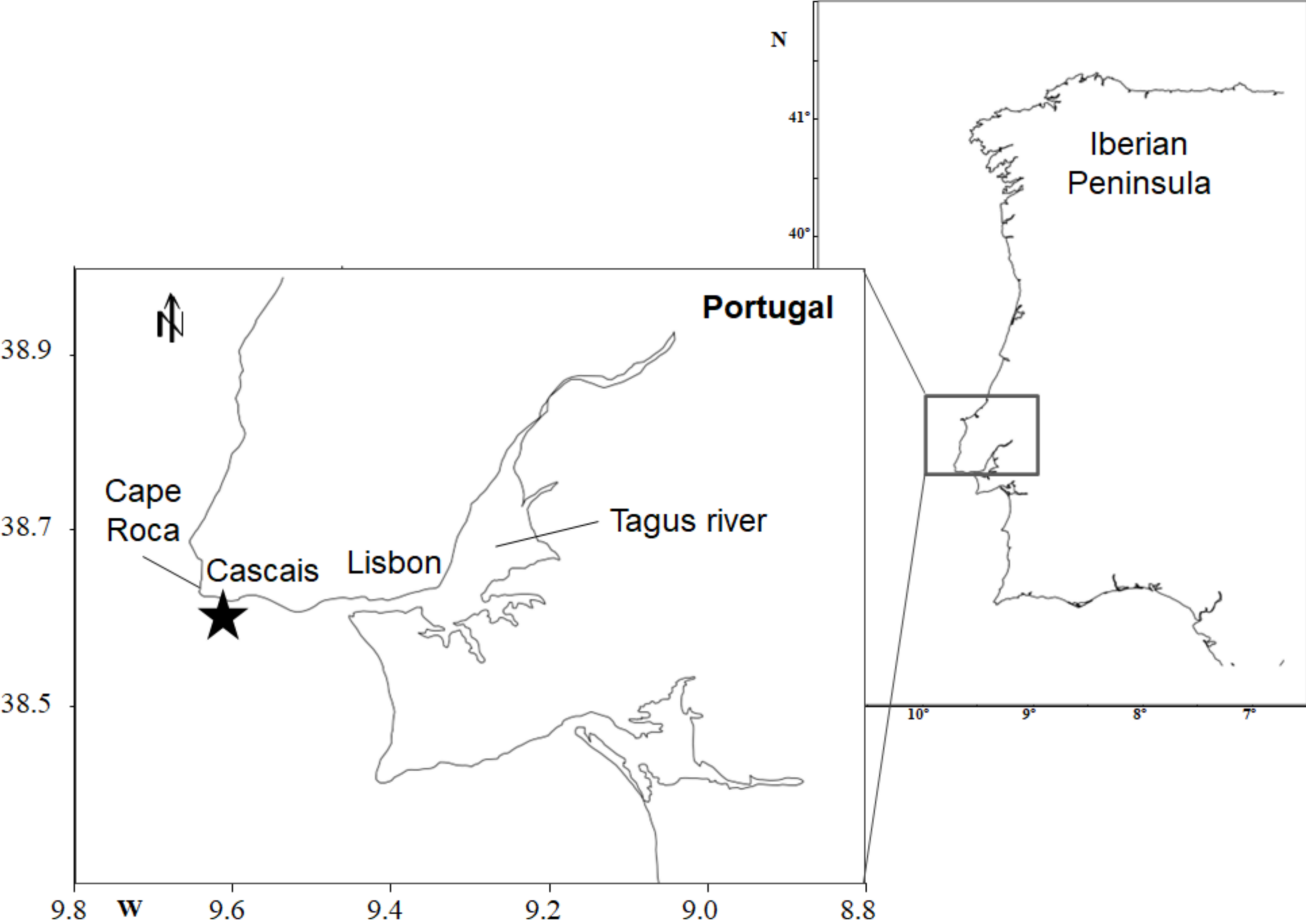
Sampling site location. Western coast of Portugal, indicating the location of Cascais Watch monitoring site (black star).

A zooplankton monitoring program has been carried out at the CCW station, off the coast of Portugal, since 2005. The sampling station is located around 2.5 nautical miles off Cascais, at 36 m depth and near the Tagus River mouth (38°40′N, 09°26.2′W) (Fig. 1). Whenever possible, samples were collected monthly, between February 2005 and June 2015 with some gaps, such as the absence of samplings for 2011 and 2012, and the limited sampling frequency from 2009 onward (four samples collected in 2009; one to two samples from 2010 onward, with no sampling during winter seasons). To reduce the influence of the Tagus River, samples were collected circa 2 h before the high tide. Samples collected 4 h before and 3 h after high tide were not considered in the data analysis. A total of 49 samples were examined.

Zooplankton was sampled with a WP-2 plankton net (0.56 m diameter, 200 µm mesh size) fitted with a flowmeter, towed obliquely between the surface and 5 m above the bottom. Each sample represented on average 48.9 ± 18.2 m^3^ of filtered sea water. Samples were immediately preserved with 4% borax buffered formaldehyde, prepared using seawater.

Vertical profiles of temperature, salinity and fluorescence were also registered with a CTD SBE 19p and a Chelsea Instruments fluorometer, deployed right before the biological sampling. However, due to the irregularity in the acquisition of these environmental data, we chose not to use them in the present analyses. Satellite-derived data of sea surface temperature (SST) and chlorophyll *a* surface concentration (Chl *a*) for the years 2005 to 2015 were obtained from MODIS ocean color products distributed through http://oceancolor.gsfc.nasa.gov/ and using SeaDAS program, Version 7.3.1. The upwelling index (2005–2015) from Cape Roca was obtained from http://www.indicedeafloramiento.ieo.es/HCRoca/ (FNMOC 6 h 1-degree Transports model; center position: 38.5N, 9.5W), while daily and monthly precipitation (2005–2015) was acquired from the Sistema Nacional de Informação de Recursos Hídricos (SNIRH, Monte da Caparica station) and considered as a proxy for river runoff and salinity.

In the laboratory, the biovolume was determined by displacement volume, which was afterwards converted to dry weight according to the equations on Wiebe (1988). Zooplankton samples were fractionated with a Folsom plankton splitter, and the identification of the specimens was conducted in the smallest fraction, totaling at least 500 organisms for the common taxa, although all fractions were searched for the less abundant ones. The identification of the specimens was made to the lowest possible taxonomic level. Abundance values were expressed as the number of individuals per cubic meter (ind.m^−3^).

### Statistical analysis

Average abundance and biomass values were tested for differences among months, seasons (hereafter referring to astronomical seasons) and years using one-factor fixed effects ANOVA models, when the homogeneity of variance was verified in the Levene test. For non-significant homogeneity of variance, Kruskal-Wallis analyses were conducted instead. Pairwise tests (Tukey or Mann–Whitney) were used to identify the factors contributing to the statistical differences. The grouping of the data intended to reflect the irregularity in the sampling effort, by using the most uniform points of comparison possible. For all the statistical analyses, taxa, when necessary, were considered by major groups (Mollusca, Diplostraca, Decapoda, *etc*.). To uncover the dominant taxa/species responsible for the observed differences in the community composition, a principal component analysis (PCA) was performed. Additional PCA analyses were performed with standardized data (zero mean and unit variance, *i.e.,* dimensionless data) to examine correlations among the environmental (temperature, chlorophyll *a* concentration, upwelling index and precipitation) and biological (biomass, abundance of zooplankton and taxa groups) parameters. The abundance ratios of Holoplankton:Meroplankton, Gelatinous:Crustaceans and Cyclopoida:Calanoida were determined to assess potential shifts in the zooplankton communities. Diversity (Shannon-Wiener; Simpson diversity index, 1-D), species richness (Margalef), eveness (Pielou, J) and taxonomic (Menhinick, D) indices were computed. Significant relationships were further explored with generalized linear models (GLM). For monthly analyses, environmental missing values were replaced by 10-year averages. Lomb-Scargle periodograms and Mann-Kendall trend tests were used on the abundance data to detect periodicities and monotonic tendencies in the occurrence patterns of each taxon, respectively. These methods are useful to explore unevenly sampled data and were applied to values of averaged abundances, adjusted by season and month, and to the entire time-series (excluding non-sampling periods). The statistical analyses were performed using Statistica (StatSoft, Inc., http://www.statsoft.com), PAST (Paleontological statistics software package for education and data analysis) (Hammer, Harper & Ryan, 2001) and MatLab (http://www.mathworks.com/).

Complementarily, dynamic factor analysis (DFA) computations were applied to the abundance data of the most abundant taxa identified in the previous analyses, using the Brodgar (Highland Statistics Ltd) software. DFA is an adequate tool to explore patterns in time-series, especially those covering short temporal periods or composed of nonstationary data (Zuur et al., 2003). Although the temporal range of the present data is not the ideal, the missing gaps on the data were one of the main reasons for the application of this technique. Nevertheless, caution is needed when examining these patterns that represent only indications that need to be improved in the future. The method provides smoothed functions through time, trends and relationships between variables. The analyses were performed on transformed data (ln [*x* + 1]) and the missing data identified as such. The environmental factors were used as explanatory variables. The DFA model fit was applied to the entire time-series and specifically to several Copepoda taxa/species. One to three common trends (CTs) were tested, and the best solution was chosen according to the lowest Akaike information criterion (AIC).

## Results

### Hydrographic conditions

**Figure 2:**
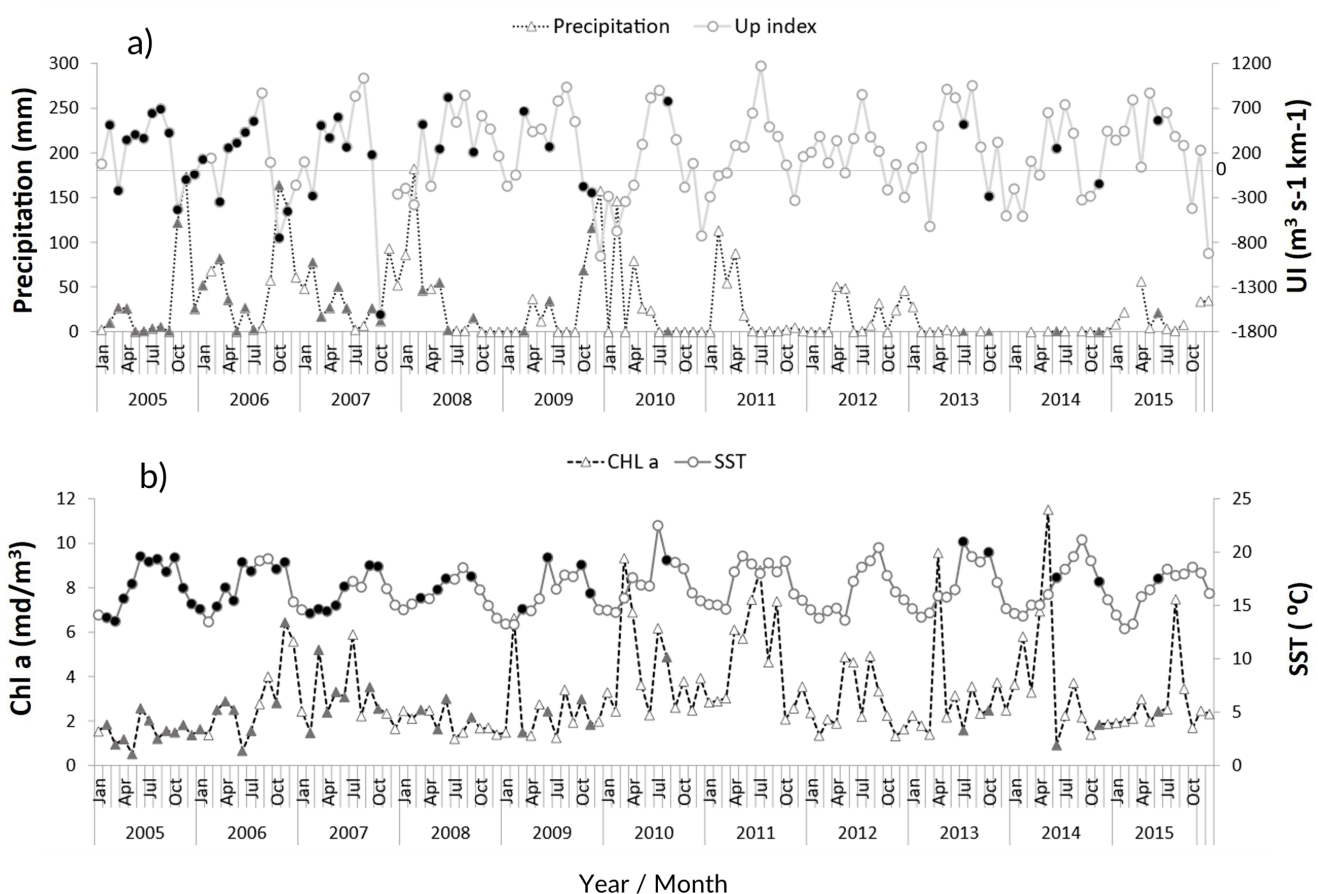
Environmental variables. Interannual monthly mean variation of the environmental parameters: (A) Precipitation and upwelling index (UI); (B) chlorophyll *a* concentration (Chl a) and Sea Surface Temperature (SST). The filled symbols correspond to months when at least one sampling was performed.

The zooplankton community at the CCW was influenced by the seasonal conditions usually found in temperate regions: high sea surface temperatures during summer and early autumn (average values above 18.1 °C from June to November), and lower temperatures in late autumn and winter (minimum of 13.5 ± 1.2 °C, Fig. 2B). Spring and summer months were also characterized by high productivity (Fig. 2B), as well as intense upwelling, which was strongest during July and August (Fig. 2A). The lowest productivity was detected in winter months, associated with the decrease of the upwelling intensity. The recent years were characterized by slightly higher maximum sea surface temperatures and increased variability in the productivity pattern (Fig. 2B). From 2011 onward, extended dry seasons were evident from the precipitation patterns, contrasting with the years prior, characterized by rainy periods from October to March and dry seasons mainly limited to late spring and summer (Fig. 2A).

### Zooplankton composition and temporal distribution

**Figure 3:**
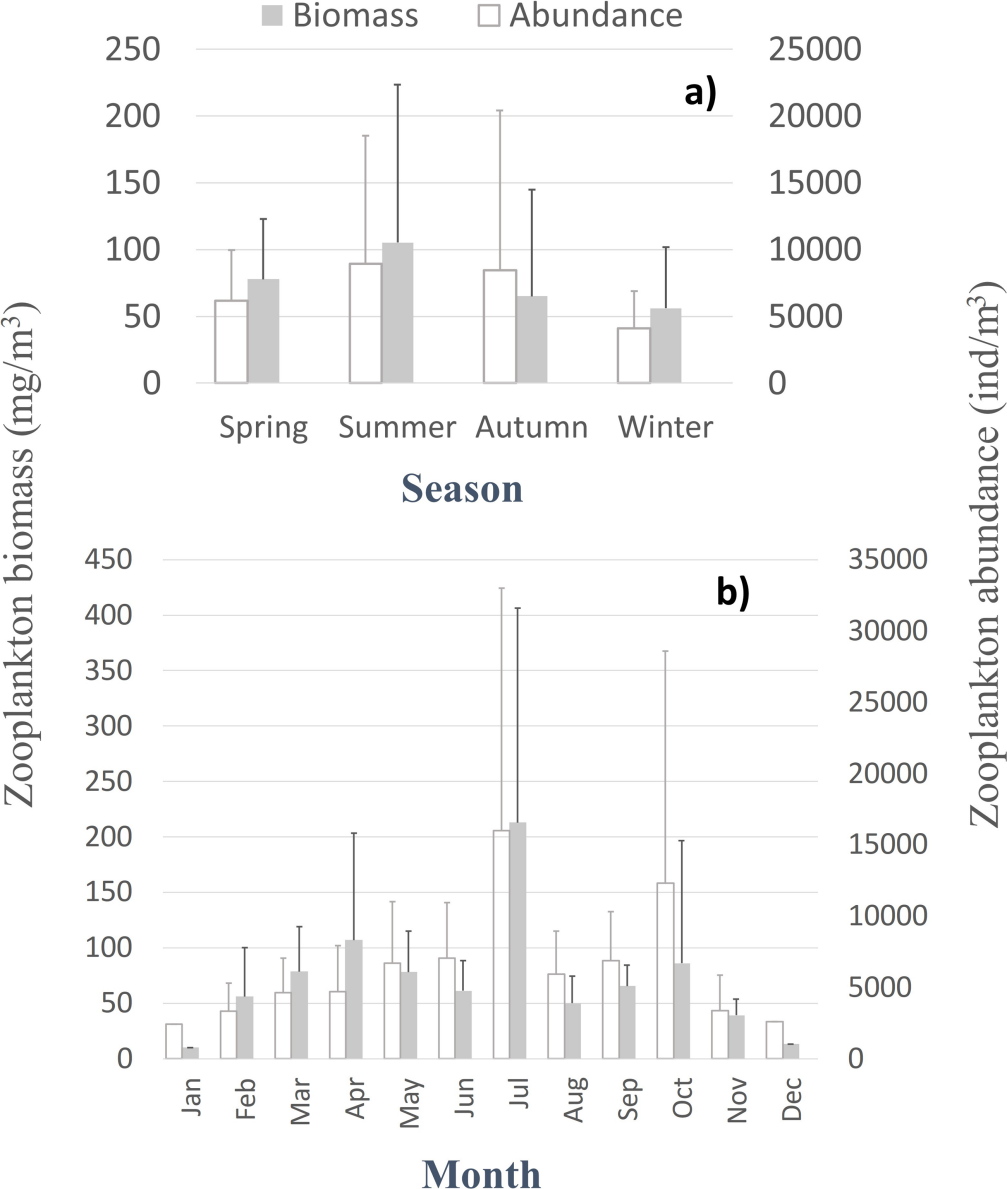
Zooplankton biomass and abundance. Average variation of the zooplankton abundance and biomass at CCW by season (A) and month (B).

Despite the seasonal patterns perceived in the environmental characterization, no significant differences, monotonic tendencies, or periodicities were detected by month or season (Tables SI and SII) in the patterns of the zooplankton abundance, biomass, and diversity at the CCW station, represented in Fig. 3 and Fig. S1. The only exceptions were the significant differences found in the zooplankton abundance for the summer and autumn samples, for the first years of sampling (Table SII). Accordingly, the monthly and seasonal patterns were relevant, with high zooplankton abundance and biomass during summer and early autumn months (Fig. 3), especially in July and October (monthly averages of 8,956 ± 11,981 ind.m^−3^, 213 ± 193.4 mg m^−3^ and 12,321 ± 16,283 ind.m^−3^, 86.2 ± 110.4 mg m^−3^, respectively). Therefore, slightly higher biodiversity was detected in summer months (Fig. S1), although not statistically significant (Table SII). The zooplankton biomass also peaked in spring (average of 77.8 ± 45 mg m^−3^, Fig. 3A), particularly in April (average of 107 ± 96.4 mg m^−3^; Fig. 3B). Winter months corresponded to the lowest zooplankton abundances (average of 4,116 ± 2,769 ind.m^−3^) and biomass (56 ± 46 mg m^−3^; 10.2 mg m^−3^in January) (Fig. 3A).

**Figure 4:**
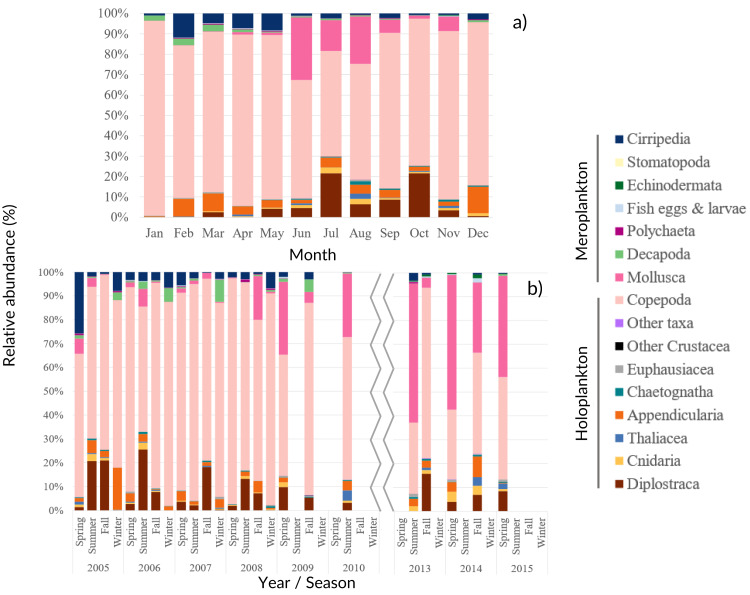
Zooplankton relative abundance. Average (A) monthly and (B) interannual seasonal variation of the taxa composition (relative abundance to the total of the samples for each period) of zooplankton at CCW. The group “Other Crustacea” includes Ostracoda, Mysida, Amphipoda, Isopoda and Cumacea, and “Other taxa” includes Ctenophora, Phronida, Foraminifera and Radiozoa.

Among the 86 taxa found, 43 were identified to family or higher level, 25 to genus and 18 to species (Table SIV). The monthly, seasonal and interannual composition of the major taxa found in the samples is presented in Fig. 4.

**Figure 5:**
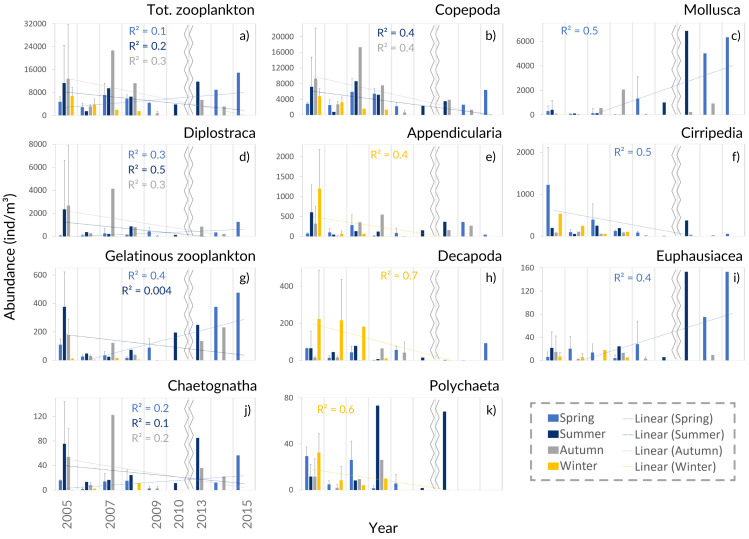
Zooplankton interannual abundance. Interannual seasonal average for the abundance of the main groups of taxa collected in the Cascais-Watch site for the entire period of 2005 to 2015. (G) Gelatinous zooplankton include the Cnidaria, Ctenophora and Thaliacea taxa. Only the most informative linear regressions are presented.

Copepoda dominated the community (71%–96% of the total), presenting two abundance peaks in July and October. The Copepoda proportion decreased in summer (Figs. 4 and 5B), following the increased contribution of Mollusca (up to circa 25–58% of the samples), Diplostraca (Cladocera) and Cnidaria (Figs. 4 and 5). Cirripedia, Appendicularia and Decapoda were a relevant component of the winter and spring samples (circa 20%).

From the PCA analyses we found that the major contributors for the observed variability in the community composition were *Acartia* spp., *Calanus* spp., *Oncaea* spp., Bivalvia, *Oithona* spp., *Evadne* spp., *Penilia avirostris* and *Centropages* spp. (Fig. S5, Table SIV). Bivalvia, *Acartia* spp. and *Evadne* spp. were associated with the summer samples, while *Penilia avirostris*, *Calanus* spp., *Oithona* spp., *Oncaea* spp. and *Centropages* spp. contributed to the distinction of the autumn samples (Fig. S5, Table SIV).

### Zooplankton interannual variability

The interannual data, showed a relative increase of the average zooplankton abundance (Fig. 5A). When comparing the years of 2005–2009 with the latter ones, a lower proportion of Copepoda, Decapoda and Cirripedia, and a higher proportion of Mollusca (mostly Bivalvia, see Table SIV) were suggested in the relative abundance (Fig. 4B). The DFA analysis also hinted at an increasing tendency of Mollusca abundance from 2011 onwards (Fig. S2A), similar abundances through the years for Copepoda, despite the decreases in 2009 and 2014, and decreases of Cirripedia and Decapoda larvae (Fig. S2).

The average meroplankton/holoplankton ratio has increased since 2009 (Fig. S4), reflecting the increase in the proportion of Bivalvia larvae. Regarding the gelatinous/Crustacea ratio, the Crustacea had a higher contribution to the samples than gelatinous zooplankton (Fig. S4), despite the enhanced abundance of the latter in spring months since 2010 (Table SIV, Fig. 5G, Figs. S2–S3). Lastly, the Cyclopoida/Calanoida ratio shows a different pattern, with low values registered in 2013–2015 (Fig. S4).

In the DFA model fit (Figs. S2 and S3), the AIC values obtained for one, two and three common trends (CTs) were, respectively: 1596.6, 1535.3 and 1509.4, for the entire time-series; and 1500.4, 1364.5 and 1317.8, for Copepoda (models with three CTs chosen as the best fit). The models generally depicted increasing trends in the abundance of Mollusca, Cnidaria and Diplostraca, but also of fish larvae/eggs and the total zooplankton abundance.

**Figure 6:**
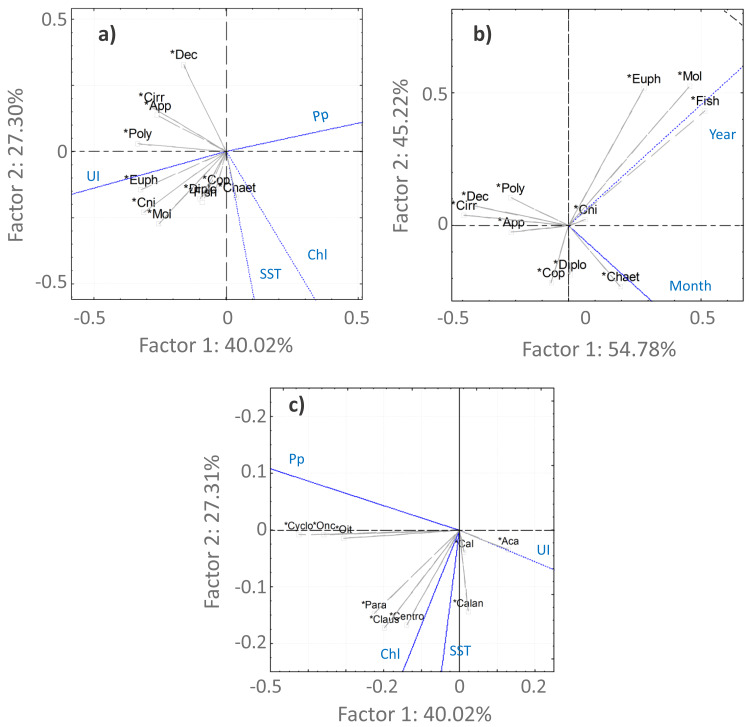
PCA analysis. Principal component analysis results for the main taxonomic groups of zooplankton (A, B) and Copepoda (C) that contributed most to the differentiation of the samples according to the environmental factors (A, C)—upwelling index (UI), precipitation (Pp), Sea Surface Temperature (SST) and chlorophyl *a* (Chl)—and temporal periods (B). For the temporal analyses, only the variable month was represented, considering the high correlation with season. The first two components are represented, accounting for more than 67% of the cumulative variance. Groups represented in the plots: abundances of Copepoda (Cop), Mollusca (Mol), Diplostraca (Dipl), Cnidaria (Cni), Appendicularia (App), Cirripedia (Cirri), Decapoda (Dec), Chaetognatha (Chaet), Polychaeta (Poly), Euphausiacea (Euph), fish eggs/larvae (Fish), Calanoida (Calan), Cyclopoida (Cyclo), *Acartia* spp. (Aca), *Calanus* spp. (Cal), *Oncaea* spp. (Onc), *Oithona* spp. (Oit), *Centropages* spp. (Cent), *Paracalanus* spp. (Para) and *Clausocalanus* spp. (Claus).

For the Copepoda, no significant monotonic tendencies were detected (Table SI), despite an apparent interannual decrease (Fig. 5B). The statistical analyses showed significant differences in copepod abundance for the period 2005–2009 (Table SII). The copepod abundance was positively correlated with the SST and the upwelling index in the PCA analyses (Figs. 6A and 6B).

**Figure 7:**
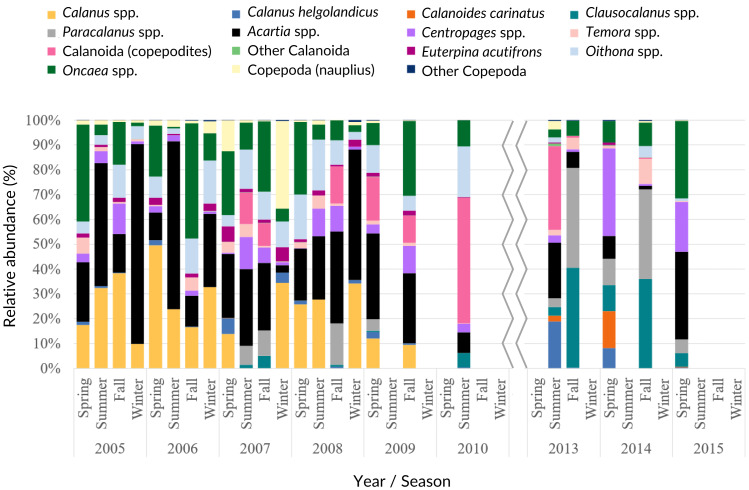
Copepoda interannual abundance. Average interannual seasonal variation (relative abundance to the total of the samples for each period) of the Copepoda composition at the CCW.

*Acartia* spp. (20% of all samples), *Calanus* spp. (14%), *Oncaea* spp. (12%) and *Oithona* spp. (6%) dominated the 2005–2009 samples (from 51 to 96%), decreasing in proportion from 2010 onwards (Fig. 7, Table SIV). In the ANOVA and Kruskal-Wallis analyses, these interannual differences were only significant for *Acartia* spp. (Table SII), although a significant monotonic decreasing tendency was detected for *Calanus* spp. in the Mann-Kendall tests (Table SI) and represented in the DFA analyses (Figs. S2 and S3). Higher proportions of Calanoida were detected in recent years, with increasing tendencies of *Clausocalanus* spp., *Paracalanus* spp. (up to 3% of the total, only significant for the former, per year; Tables SI, SII and SIV), and *Centropages* spp. (Fig. 7), also generated by the DFA analyses (Figs. S2 and S3). In the PCA analyses, *Calanus* spp., *Acartia* spp. and *Paracalanus* spp. showed positive correlations with the SST (Fig. 6C).

The observed decrease in the Cyclopoida: Calanoida ratio, with lower proportions of Cyclopoida in the 2013–2014 samples (Table SIV, Fig. S4), followed the decreasing trend of *Oithona* spp. shown in the DFA models (Figs. S2B, S2E, S2F and S3B). On the other hand, the lower proportion of *Oithona* spp. was positively correlated with precipitation, as shown in the PCA analyses (Fig. 6C). A positive correlation with precipitation was also obtained for *Oncaea* spp. (Fig. 6C). Additionally, *Oithona* spp. was positively correlated with the upwelling index in the GLM analyses (Table SV).

The increasing monotonic tendency of Mollusca (circa 10% in the total samples; Table SI), was significantly different for late spring and summer months (Fig. 5C), with positive correlations with year, upwelling intensity, and SST in the PCAs (Figs. 6A and 6B). Bivalvia, the most represented (average of 97% of the samples; Table SIV), showed a monotonic increasing tendency per season and month (MK *p* < 0.02 in all cases; not shown), and a significant correlation with SST (Table SV). For Mollusca, significant differences were found between the first and later years of sampling (Table SII).

Abundance peaks of Diplostraca (Cladocera) were detected in July and October (Fig. 4A). No monotonic tendencies were observed (Table SI), despite the apparent autumn and summer interannual decrease (Fig. 5D). The slight increase for spring periods (Figs. 4B and 5D) was also hinted at in the DFA analyses (Figs. S2 and S3). Positive PCA-based correlations were obtained with SST and the upwelling intensity (Fig. 6A). *Evadne* spp. and *Penilia avirostris* were the most abundant taxa (48 and 43% of the total, respectively) (Table SIV) and the latter showed significant correlations with SST and the upwelling intensity (Table SV).

Cirripedia registered the highest abundances in late winter and spring (Fig. 4A), with monotonic decreasing tendencies (Table SI, Fig. 5F), that were not entirely represented in the DFA analyses (Figs. S2 and S3). The PCA analyses revealed negative correlations with SST and chlorophyll *a* (Fig. 6A).

For Cnidaria, mainly siphonophores (circa 60%) and hydromedusae (39%) were identified (see Table SIV). July registered the highest abundances (Fig. 4A) and, the apparent spring/autumn interannual increase (Figs. 4B and 5G), depicted in the DFA analyses (Figs. S2 and S3), did not manifest in monotonic tendencies (Table SI). Positive PCA-based correlations were obtained with year, SST and upwelling intensity (Figs. 6A and 6B).

Decapoda showed decreasing monotonic tendencies (Table SI), and significant differences for winter samples and the first year of sampling (Table SII). Abundance peaks were detected in winter and spring (Fig. 4B), as well as March (Fig. 4A), showing a significant correlation with SST in the GLM analyses (Table SV).

## Discussion

The study presents a relevant analysis of the information available for the seasonal and interannual variability of the zooplankton community in the CCW coastal station from 2005 to 2015 and their potential long-term changes. Given the scarcity of recent samples, the patterns must be carefully considered. Nevertheless, given the lack of knowledge for the region, the analyses highlight some shifts in the local community that need to be verified and monitored in the following years through the maintenance and the increase of the sampling frequency in the CCW.

### General patterns of zooplankton biomass, abundance and composition

High year-round productivity was observed for the study area, without major significant trends or periodicities detected in the zooplankton abundance, biomass, and diversity, even despite the relatively lower winter values. The high chlorophyll levels during the entire year were undeniable, when compared with values obtained in other works (*e.g.*, Bode et al., 2009; Eloire et al., 2010; Bresnan et al., 2015; Yebra et al., 2020), namely for Galician waters (Buttay et al., 2016). The average zooplankton biomass followed the values reported by Domínguez et al. (2017) for the northern Portuguese coast. Nevertheless, the present study detected spring/summer and autumn peaks in zooplankton abundance, complying with the common seasonal shifts reported for zooplankton communities of temperate latitudes and for the region (*e.g.*, Valdés & Moral, 1998).

The composition of the zooplankton community at CCW was alike to what has been reported for the Iberian coast (*e.g.*, Valdes et al., 1990; Valdés & Moral, 1998; Valdés et al., 2007; Bode et al., 2009; Sobrinho-Gonçalves et al., 2013; Domínguez et al., 2017). The dominance of Copepoda also followed the results of works for other areas of the northeastern Atlantic and adjacent seas (*e.g.*, Eloire et al., 2010; Bresnan et al., 2015; Valdés et al., 2021). The spring/summer decrease observed in the Copepoda proportion was not accompanied by the taxa abundance, which was higher during these periods, largely due to the greater diversity of other zooplankton groups and the dominance of certain copepod taxa. *Acartia* spp., *Paracalanus* spp., *Clausocalanus* spp., and *Oncaea* spp., highly abundant in the samples, have also been described as widely distributed and frequent in the northwestern Iberian coast (*e.g.*, Bode et al., 2012), being some of the most common taxa recorded in the northeastern Atlantic (*e.g.*, Valdés et al., 2007; Sobrinho-Gonçalves et al., 2013; Domínguez et al., 2017). The importance of Copepoda as intermediaries between distinct trophic levels across the marine food web is undeniable, namely for planktivorous fish (*e.g.*, Garrido et al., 2015) that usually decrease in abundance following the lower availability of Copepoda (*e.g.*, Heneghan et al., 2020).

### Interannual shifts in the zooplankton community

The spring/summer and autumn zooplankton abundance peaks followed the seasonal variation of the environmental variables, namely the sea surface temperature and upwelling index, which had relevant impacts in some of the taxa (see below). The link between the zooplankton abundance and the upwelling intensity is not surprising, given its well-known importance for zooplankton productivity (*e.g.*, Pires & Dos Santos, 2020). In the Portuguese coast, wind-driven upwelling of colder and richer deep waters is frequent in spring/summer months, when temperature increases and north/northwestern winds and southward currents dominate, contrasting with the winter prevalence of southwestern winds and northward slope flows (*e.g.*, Relvas et al., 2007). The upwelling regimes, together with the influence of the Tagus plume, especially when coupled with high precipitation (*e.g.*, Vaz et al., 2009), enhance chlorophyll concentration and phytoplankton production at surface coastal waters, leading to increased zooplankton abundance. The local sheltered dynamics, with slower currents and recirculation features, result in the coastal retention of plankton (*e.g.*, Moita et al., 2003) thus contributing to the high productivity and biodiversity observed herein. Indeed, previous works pointed to distinct inshore and offshore communities of zooplankton in the Portuguese coast (*e.g.*, Bartilotti et al., 2014; Domínguez et al., 2017), stressing the importance of the coastal environmental conditions in shaping zooplankton communities. This may have important implications for the regional ecosystem yield capacity, since the frequent local generation of upwelling filaments (*e.g.*, Haynes, Barton & Pilling, 1993) may contribute to the transfer of productivity towards offshore areas.

The slightly increasing interannual zooplankton abundances detected herein for spring periods, contrast with the general declining trends reported for the North Atlantic (*e.g.*, Iriarte et al., 2022; McQuatters-Gollop et al., 2022). Nonetheless, our observations parallel the increasing zooplankton trends detected for the Benguela upwelling region (Verheye & Richardson, 1998). Inhabiting a transition area between temperate and tropical environments (*e.g.*, Boaventura et al., 2002) that comprise the northern/southern limit of distribution of many species, the CCW zooplankton communities may experience less drastic effects than those reported for the North Atlantic. On the other hand, the upwelling regimes, as one of the drivers of long-term increase of crustacean zooplankton (Verheye & Richardson, 1998), may also explain the observed trend. In the long-term analyses for the western Atlantic by Morse et al. (2017), the spring community shifts detected in the composition and abundance of zooplankton were associated with environmental changes mediated by the North Atlantic Oscillation (NAO).

We report two main periods (2006 and 2009/2010) of lower abundance of zooplankton, particularly for Copepoda, fish larvae, Cnidaria, Diplostraca and Cirripedia, that apparently induced shifts in the community. The 2009/2010 period was characterized by unusual climatic variability throughout the North Atlantic, including warmer and more saline waters, driven by a strong negative NAO (*e.g.*, Hughes, Holliday & Beszczynska-Möller, 2011). During this period, in Portugal, heatwaves were registered from spring to autumn, extending the annual dry season (IPMA, 2010; IPMA, 2011).

#### Copepoda

Concerning the Copepoda, our results suggest that the periods 2006 and 2009/2010 may mark changes in species composition and dominance of the most abundant taxa, namely lower abundances of *Acartia* spp. and *Calanus* spp., and increased abundances of *Paracalanus* spp. and *Clausocalanus* spp.. Similar results, particularly the replacement of *Acartia* species, dominant in spring, by *Paracalanus* spp., highly abundant in summer, were obtained by Iriarte et al. (2022) for the Bay of Biscay. These and other authors (*e.g.*, Buttay et al., 2016; Dessier et al., 2018) reported points of regime shifts and high variability for the Copepoda species composition in the northeastern Atlantic throughout the 2002–2018 year span, particularly 2006 and 2010.

The decrease in proportion of larger Copepoda such as *Calanus* spp. is an important shift that may impact the community structure (Barton et al., 2013), as well as the trophic links and the energy transfer through the food web (Heneghan et al., 2020), influencing for example the regimes of planktivorous fishes (Verheye & Richardson, 1998). Northward expansions of the distribution limits of *Calanus helgolandicus* in the north Atlantic associated with climate variability and driven by warmer southern currents have been suggested (*e.g.*, Bonnet et al., 2005; Bode et al., 2009).

*Acartia* spp., typical inhabitants of estuarine and coastal areas (*e.g.*, Bode et al., 2005), were the most abundant Copepoda. Although no significant trends were detected, the apparent interannual decrease may represent changes both in the food web and the environmental conditions. Impacts in the trophic transfer were previously suggested for *Acartia tonsa* in a long-term monitoring for Chesapeake Bay (Kimmel, Boynton & Roman, 2012), given the importance of the taxa for planktivorous fish. The location of CCW, at the mouth of the Tagus River, may explain the abundance of the taxa. Previous studies suggested that *Acartia* species have a diverse range of prey (Bollens & Penry, 2003), adapting their feeding preferences according to the oceanic conditions (Kiørboe, Saiz & Viitasalo, 1996). Moreover, previous evidence showed that climate change may promote earlier peaks in the occurrence of zooplankton, as reported for some Copepoda and meroplankton (*e.g.*, Edwards & Richardson, 2004).

Our results suggest a strong link between the abundance of Copepoda and the environmental factors, given the correlation with SST and the upwelling intensity. The relative interannual decrease of Copepoda reported herein is mainly accompanied by the lower proportion of Cyclopoida, such as *Oithona* spp. in recent years, against the enhanced abundance of Calanoida species (*Clausocalanus*, *Paracalanus* and *Centropages*). This shift in the abundance of copepod taxa composition, namely the decrease of *Oithona* spp., is also related with the extension of the dry season discussed above, as shown by the positive correlation with precipitation. The effect of warmer temperatures and negative correlations with SST were previously reported for *Oithona similis* (Castellani et al., 2016). Similarly, the observed interannual decrease of *Oncaea* spp. from 2010 onwards, followed the correlation found with precipitation, especially considering that the recent years were characterized by warmer and dry periods, with the occurrence of frequent heat waves. As follows, *Oncaea* spp. dominated the autumn period, which can also be related with the decreased relative abundance of *Acartia* and *Paracalanus*/*Clausocalanus* species. *Oncaea* spp. are widely distributed in the northeastern Atlantic (Valdés et al., 2007) and are considered as indicators of coastal upwelling (Blanco-Bercial, Álvarez Marqués & Cabal, 2006). Nevertheless, in the present study they were found when the upwelling intensity was lowest. However, consistently high abundances were recorded annually, with higher seasonal values than the spring peak recorded by Sobrinho-Gonçalves et al. (2013). *Oncaea* spp. are thermophilic, omnivore and opportunistic species, adapting to warmer and more stratified conditions (Valdés et al., 2007), typical conditions in summer and autumn, when the taxa dominate the northwestern Iberian zooplankton communities (Valdes et al., 1990; Blanco-Bercial, Álvarez Marqués & Cabal, 2006), as suggested by our data.

#### Gelatinous zooplankton

The increasing importance of the gelatinous fraction (particularly Cnidaria) for spring/summer months in recent years, closely linked with SST, hint at changes in primary production and prey dynamics. The same pattern was previously reported in the region (*e.g.*, Valdes et al., 1990; D’Ambrosio et al., 2016), contrasting with autumn periods when this component is less relevant (Domínguez et al., 2017). D’Ambrosio et al. (2016) detected interannual increasing trends and earlier occurrences of gelatinous zooplankton since 2007, as reported for other areas where these patterns were linked with climate changes (*e.g.*, Molinero, Casini & Buecher, 2008). As suggested by our results, these patterns were particularly related to warmer water temperatures (*e.g.*, Purcell et al., 2012), and enhanced variability of both upwelling and NAO. Temperature is known to enhance the reproductive efficiency of many species of gelatinous zooplankton, increasing the magnitude and frequency of their occurrences (*e.g.*, Purcell et al., 2012). In addition, their ability to survive in conditions that may be detrimental to other taxa results in large outbreaks in coastal areas (Richardson, 2008). Thus, the environmental variability is regarded as the main driver of the abundance of gelatinous zooplankton, especially regarding upwelling and water temperature (*e.g.*, Lucas et al., 2014).

Siphonophores, the most abundant Cnidaria in the CCW, are avid predators of zooplankton and their abundance follows that of their preys, such as Copepoda or Diplostraca (*e.g.*, D’Ambrosio et al., 2016). Indeed, Diplostraca and the most represented of this group, *Penilia avirostris*, showed a preference for warmer waters, agreeing with what was reported by Domínguez et al. (2017) for the adjacent northern coastal area. Thus, the increased abundance of the cnidarians complies with the interannual Copepoda decrease, further highlighting the importance of trophic interactions in our study site. These trends may have important ecological implications, since gelatinous zooplankton play a key role in the food web, comprising the prey of several fishes, especially when other food resources are scarce (Brodeur et al., 2019). However, this implies lower nutritional value and growth potential for higher trophic levels, potentially affecting some ecosystem services (*e.g.*, fisheries).

#### Mollusca and other meroplankton

The higher abundance of meroplankton in recent sampling years, such as Bivalvia and fish larvae/eggs, in relation to holoplankton, suggests increased inputs of the local adult populations. The importance of the meroplankton fraction for summer zooplankton communities has been widely recognized (*e.g.*, Valdes et al., 1990; Bode et al., 2005; Blanco-Bercial, Álvarez Marqués & Cabal, 2006). In the Cascais area, rocky beaches create diverse habitats available for fishes and invertebrates (*e.g.*, Flores, Cruz & Paula, 2002). Considering the adequate conditions for larval survival, namely the access to food, promoted by the link with the Tagus River and the sheltered coastal dynamics, the increase of meroplankton may first represent periods of intense reproductive activity, driven by specific environmental conditions, reflecting seasonal successions in the community. Valdes et al. (1990) reported Bivalvia as one of the most abundant components of the inshore zooplankton community in the Galician coast for spring/summer. The correlation with the coastal upwelling may also explain the high abundance of the taxa, as it implies an enhancement of the supply of meroplankton from benthic habitats into the water column. Furthermore, the relevant abundance and interannual increasing trends of early Copepoda stages (copepodites), prey of sardine and anchovy (Morote et al., 2010), for example, may also explain the higher abundance of fish larvae. Notwithstanding, long-term increases of meroplankton have been detected in the northwestern European shelf across wide spatial scales by Bedford et al. (2020). The authors correlated the increase in meroplankton with the SST and the increased abundance of small Copepoda.

We report a conspicuous increase in the abundance of Bivalvia in recent years. Although species specific information could not be obtained from our samples, we hypothesize that this trend may be linked to the increasing abundance of the Manila clam (*Ruditapes philippinarum*) in the Tagus River region. Besides the native species, *Ruditapes decussatus*, whose abundance has declined (Garaulet, 2011), the Manila clam is one of the most frequently observed species in the Tagus River (Dias et al., 2019). The species, native from the Indo-Pacific region, dispersed throughout the European estuaries, due to human introductions for aquaculture purposes. In Portugal, despite the limited information available, the species is known to have inhabited the Tagus estuary for more than a decade (Garaulet, 2011). Two possible recruitment periods in spring–early summer and autumn–early winter were reported in other areas (Humphreys et al., 2007). Considering the lifetime of Bivalvia larvae (ca. 15–30 days reported for *R. decussatus*; Chícharo & Chícharo, 2001), the abundance peaks observed in the present study match the recruitment season of the species. In addition, *R. philippinarum* reaches gonad maturation above 18 °C (Solidoro, Canu & Rossi, 2003), temperatures registered in early summer at CCW, corroborating the possibility that this may be one of the species behind the increase. In any case, the great variety of forms and sizes of the Bivalvia detected in the samples suggests a high diversity of species within this group. Attempts to uncover the species driving the Bivalvia increase through molecular techniques are underway.

On the contrary, for the meroplanktonic crustaceans, Decapoda and Cirripedia, the decreasing trend in abundance highlighted in the analyses may indicate that the recruitment of benthic crustaceans is decreasing in the study region, contrasting with other regions where crustacean larvae have been increasing and bivalve larvae declining (*e.g.*, Kirby, Beaugrand & Lindley, 2008). The higher abundance of crustacean larvae in winter and spring agrees with the exhibited negative correlation with SST. Therefore, it is expected that crustacean larvae (decapods and cirripedes) will decrease in a scenario of increasing SST. However, the reduction of sampling effort since 2009 and the lack of data for the 2011–2012 years may be influencing the results. As follows, maintaining the CCW monitoring and increasing the sampling effort is extremely necessary.

## Conclusions

The present work adds important knowledge on the zooplankton coastal communities of the northeastern Atlantic, helping to fill the knowledge gap between the northern Iberian margin/Bay of Biscay and the Mediterranean Sea/southern Atlantic. Despite the gaps in the sampling series and the need for caution when examining the trends presented herein, the indications provided are relevant.

Contrary to what was expected, the results pointed to a year-round high productivity in the CCW station with no major significant trends or periodicities in the zooplankton abundance, biomass and diversity, although with a perceived seasonal pattern with a peak in summer/early autumn and low values in winter, following the seasonal variability in temperature. An increasing interannual zooplankton abundance was detected for spring periods, in agreement with the Benguela upwelling region (Verheye & Richardson, 1998) and contrasting with the general declining trends reported for the North Atlantic (*e.g.*, McQuatters-Gollop et al., 2022). Copepoda dominated the zooplankton community, represented mainly by small size species as *Acartia* spp., *Paracalanus* spp., *Clausocalanus* spp. and *Oncaea* spp., displaying a strong link with SST and the upwelling intensity.

The results revealed two main transition periods with marked changes in species dominance for the most abundant taxa, which were tentatively attributed to the extending annual dry seasons in Portugal after 2011, with very low values of precipitation, warmer and more saline estuarine waters. An increasing importance of the gelatinous species (particularly Cnidaria) for spring/summer months in recent years was also evident.

Another relevant tendency in recent years, was the higher abundance of meroplankton (Bivalvia and fish larvae/eggs) and the decreasing trend in the abundance of meroplanktonic coastal crustaceans (Decapoda and Cirripedia), highlighting possible changes on the benthic coastal populations in the study region and, contrasting with the scenarios found in other regions (*e.g.*, Kirby, Beaugrand & Lindley, 2008).

As zooplankton is a key component of marine coastal ecosystems, long-term time series are still the better tool to assess the dynamics of zooplankton communities in all its biological, physical, and chemical components. Monitoring of planktonic communities at the CCW needs to continue through the increasing of the sampling effort, being especially important regarding the predicted climate changes and in face of the results obtained in the present study.

##  Supplemental Information

10.7717/peerj.16387/supp-1 Figure S1 Diversity indices Average seasonal (a) and monthly (b) variation of zooplankton diversity at CCW: S-W, Shannon-Wiener diversity index; Margalef’s species richness; Simpson diversity index (1-D); Pielou’s Eveness (J); Menhinick taxonomic richness index (D).

10.7717/peerj.16387/supp-2 Figure S2 DFA results Dynamic Factor Analysis (DFA) results. Model fit and respective factor loadings (two and three common trends) applied to the most abundant taxa collected during the entire time-series for all taxa (a, c, d) and Copepoda (b, e, f), presenting the observed (black dots) and fitted (line) abundances through time in the left panels. Groups represented in the plots: zooplankton biomass (Biom), abundances of total zooplankton (TZoo), Copepoda (Cop), Mollusca (Mol), Diplostraca (Dipl), Cnidaria (Cni), Appendicularia (App), Cirripedia (Cirri), Decapoda (Dec), Chaetognatha (Chaet), Polychaeta (Poly), fish eggs/larvae (Fish), Calanoida (Calan), Cyclopoida (Cyclo), *Acartia* spp. (Aca), *Calanus* spp. (Cal), *Oncaea* spp. (Onc), *Oithona* spp. (Oit), *Centropages* spp. (Cent), *Paracalanus* spp. (Para) and *Clausocalanus* spp. (Claus).

10.7717/peerj.16387/supp-3 Figure S3 DFA results—common trends and residuals Dynamic Factor Analysis (DFA) results. Common trends and residuals (three common trends) applied to the most abundant taxa collected for the entire time-series (a) and Copepoda (b), presenting the observed (black dots) and fitted (line) abundance through time in the right panels.

10.7717/peerj.16387/supp-4 Figure S4 Interannual monthly variation of abundance ratios Interannual monthly variation of the abundance ratios of (a) gelatinous *versus* crustacean zooplankton, (b) Cyclopoida *versus* Calanoida copepods and (c) meroplankton *versus* holoplankton at the Cascais Watch site.

10.7717/peerj.16387/supp-5 Figure S5 PCA for taxa groups with season Principal Component Analysis results for the first two components (64.8% of cumulative variance), representing the taxonomic groups that contributed most to the differentiation of the samples from distinct seasons, represented by ellipses –Autumn (Au), Summer (Su), Winter (Wi), Spring (Sp): *Penilia avirostris* (penavi), *Calanus* spp. (calanu), *Oithona* spp. (oithon), *Centropages* spp. (centro), *Oncaea* spp. (oncaea), *Evadne* spp. (evadne), *Acartia* spp. (acarti), Bivalvia (bivalv).

10.7717/peerj.16387/supp-6 Table S1 Statistical results for Mann-Kendall monotonic trend tests and Lomb-Scargle periodogram Statistical results for the Mann-Kendall monotonic trend tests (left panel) and Lomb-Scargle periodogram (right panel) analyses regarding the most abundant zooplankton and Copepoda taxa in the CCW station, applied to the interannual monthly abundance values. Mann-Kendall Z values above 5% significance are highlighted in bold and indicate significant increasing (positive) or decreasing (negative) trends. For the Lomb-Scargle analyses, the frequencies of the power peaks are presented.

10.7717/peerj.16387/supp-7 Table S2 Statistical results for ANOVA and Kruskal-Wallis analyses Statistical results for the one-way ANOVA and Kruskal-Wallis analyses, and respective pairwise tests (Tukey and Mann-Whitney, respectively), for the main taxa detected in the CCW samples, the diversity measures and the environmental parameters (SST–Sea Surface Temperature, UI –upwelling index, Chl –chlorophyll *a*, Pp–precipitation) per month (Mar–March, Apr–April, Jun–June, Jul–July, Sep–September, Oct–October, Nov–November), season (Wi –winter, Sp –spring, Su –summer, Au –autumn), year and period (upwelling/downwelling). The significant tests are marked in bold and grey.

10.7717/peerj.16387/supp-8 Table S3 Statistical results for PCA Statistical results for the Principal Component Analysis, presenting the main components that explain sample variability relatively to environmental factors and taxa abundance.

10.7717/peerj.16387/supp-9 Table S4 Taxa frequency of occurrence Seasonal values of frequency of occurrence (F) and mean abundance (Ab; ind m^−3^ ± SD) for the different taxa identified in the CCW for the entire period of 2005 to 2015.

## Additional Information and Declarations

### Data Availability

The following information was supplied regarding data availability: The data is available at: Dos Santos A, Niza H, Cruz J, Santinho C, Dominguez R, Quintela MF, Pires RFT. 2020. Zooplankton monitoring of the Lisbon Bay, Portugal, since 2005. http://ipt.gbif.pt/ipt/resource?r=cascaiswatch.
