# Supplementary figures and images for: Zooplankton biodiversity and temporal dynamics (2005–2015) in a coastal station in western Portugal (Northeastern Atlantic Ocean)
Source: PeerJ. 2023 Nov 21;11:e16387. doi: 10.7717/peerj.16387 (PMC10668806; doi:10.7717/peerj.16387)

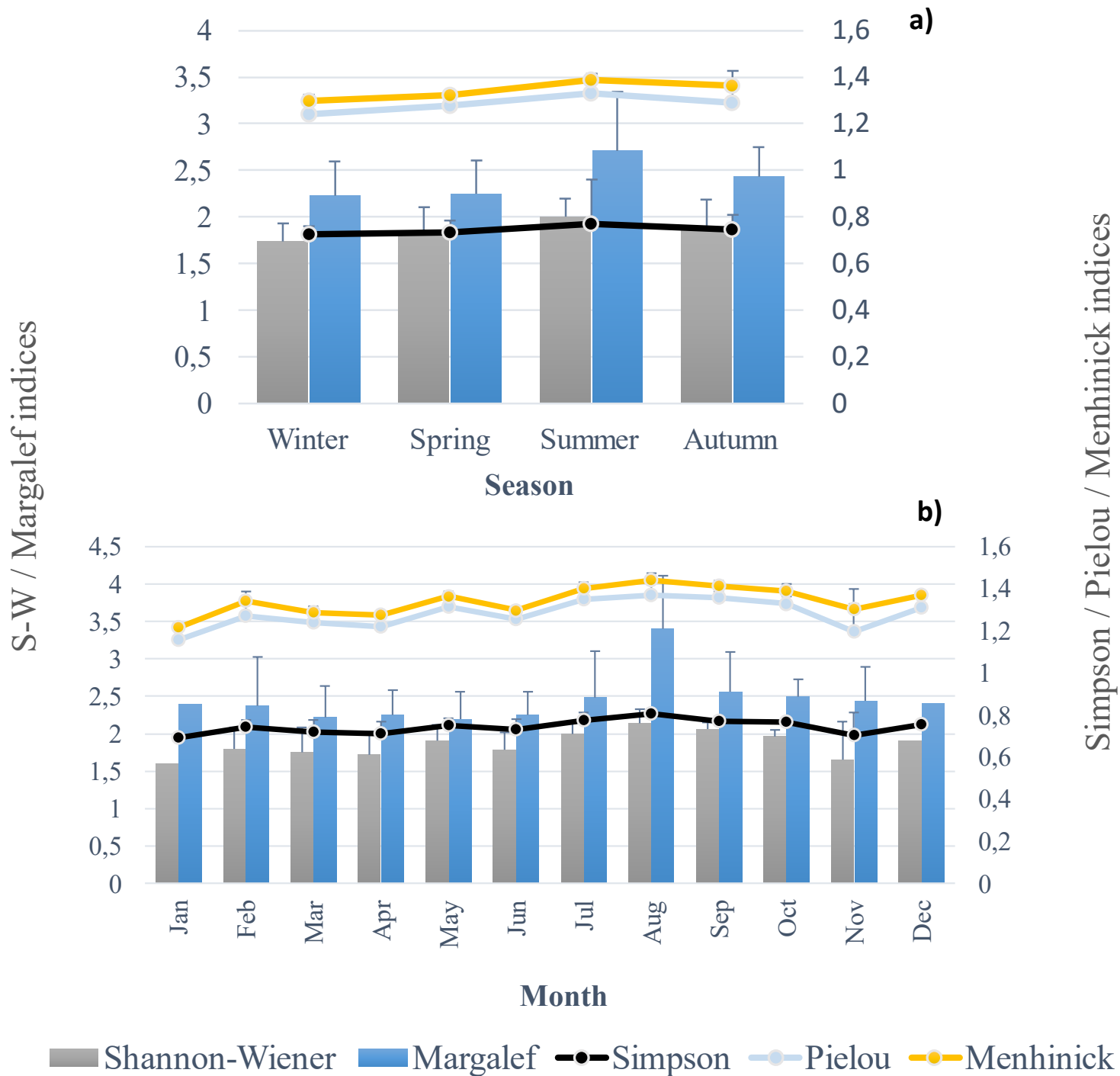

Supplement: Figure S1 — Average seasonal (a) and monthly (b) variation of zooplankton diversity at CCW: S-W, Shannon-Wiener diversity index; Margalef’s species richness; Simpson diversity index (1-D); Pielou’s Eveness (J); Menhinick taxonomic richness index (D). [file peerj-11-16387-s001.pdf]

### a) Time-series, All taxa

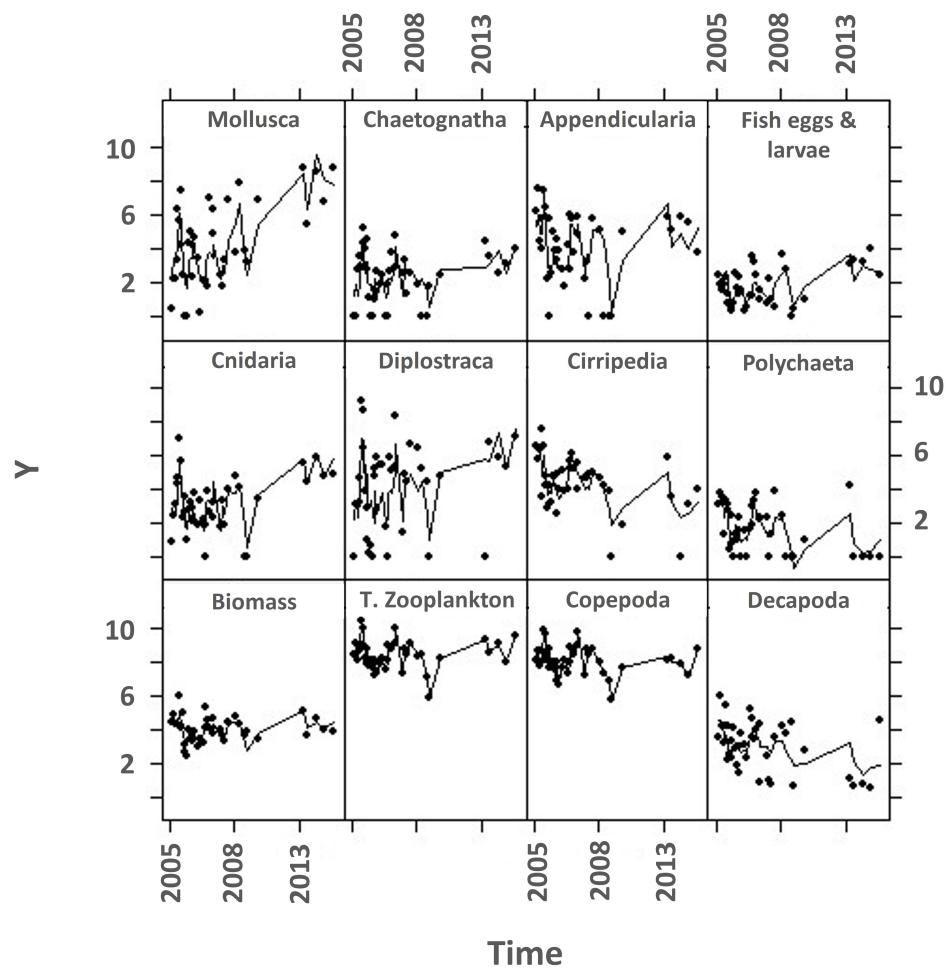

### b) Copepoda

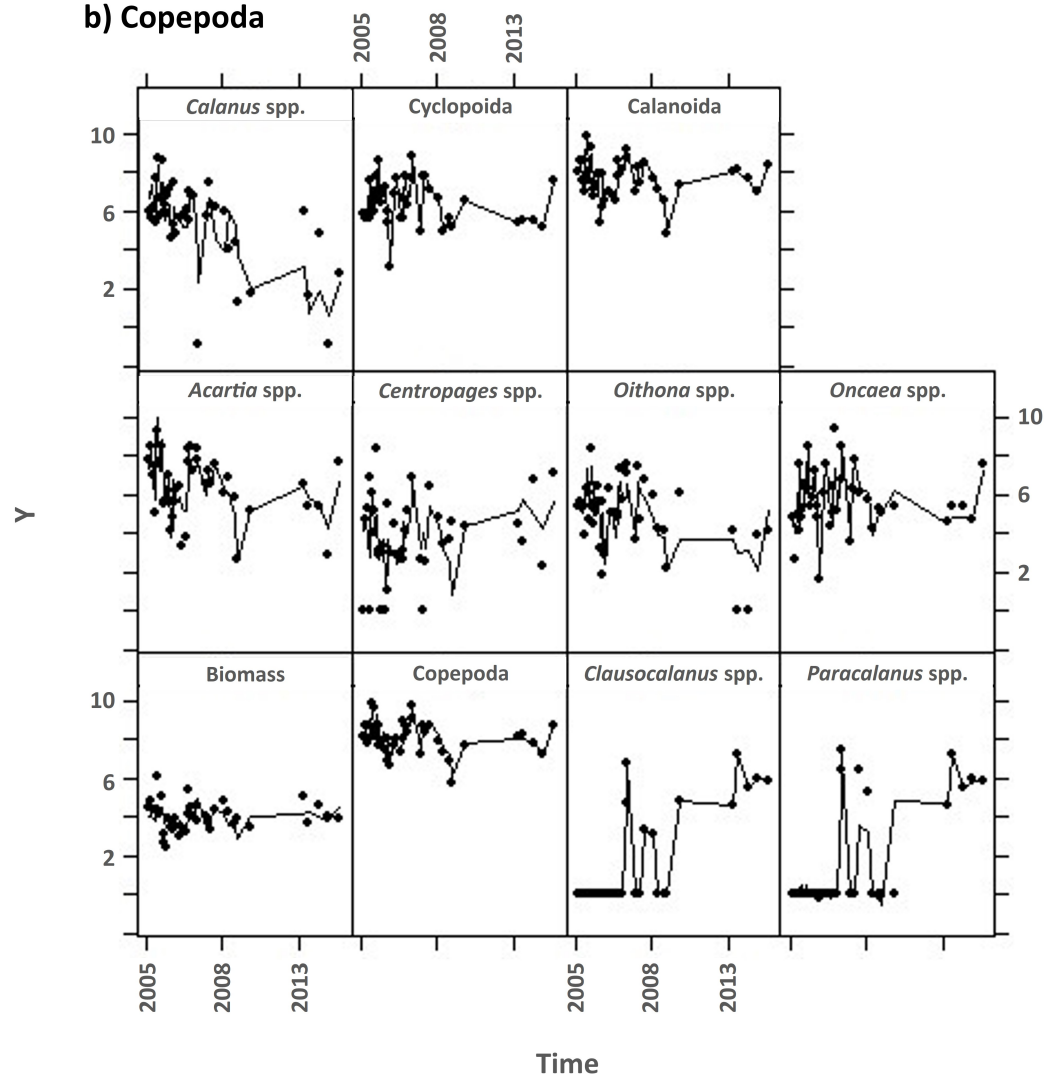

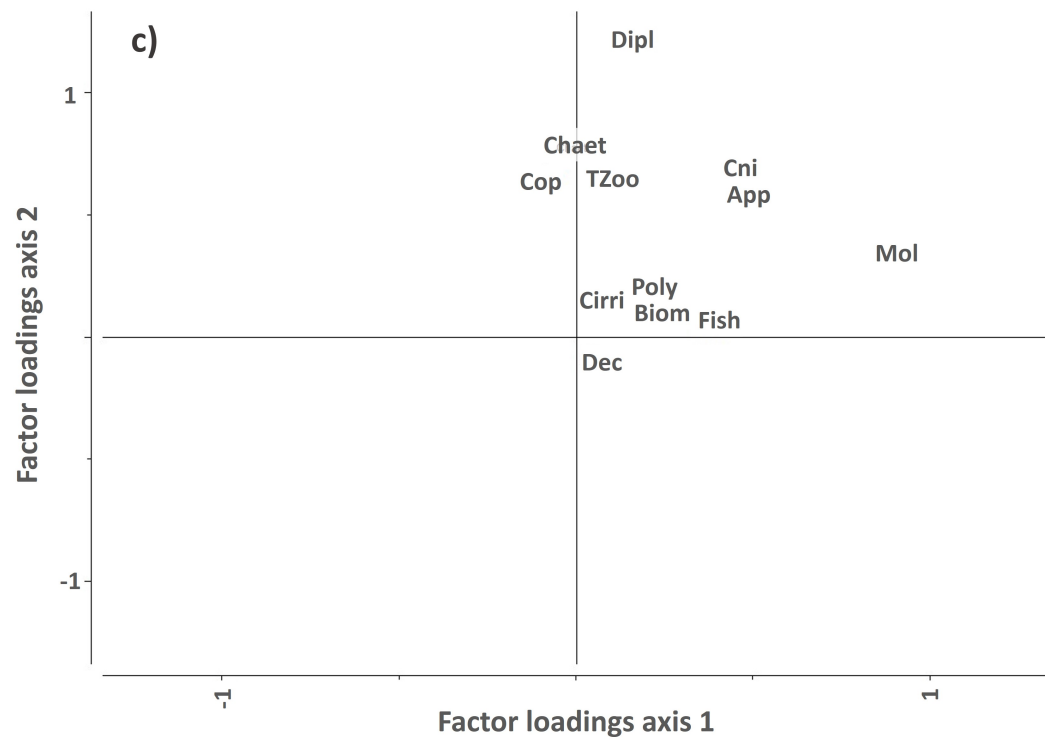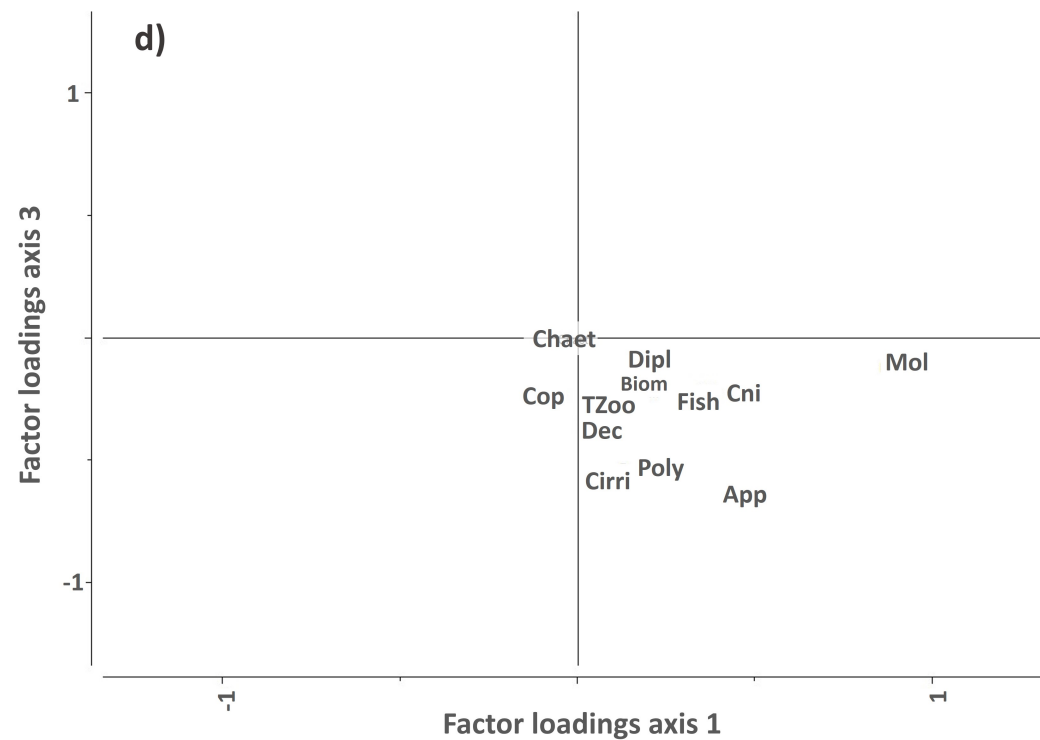

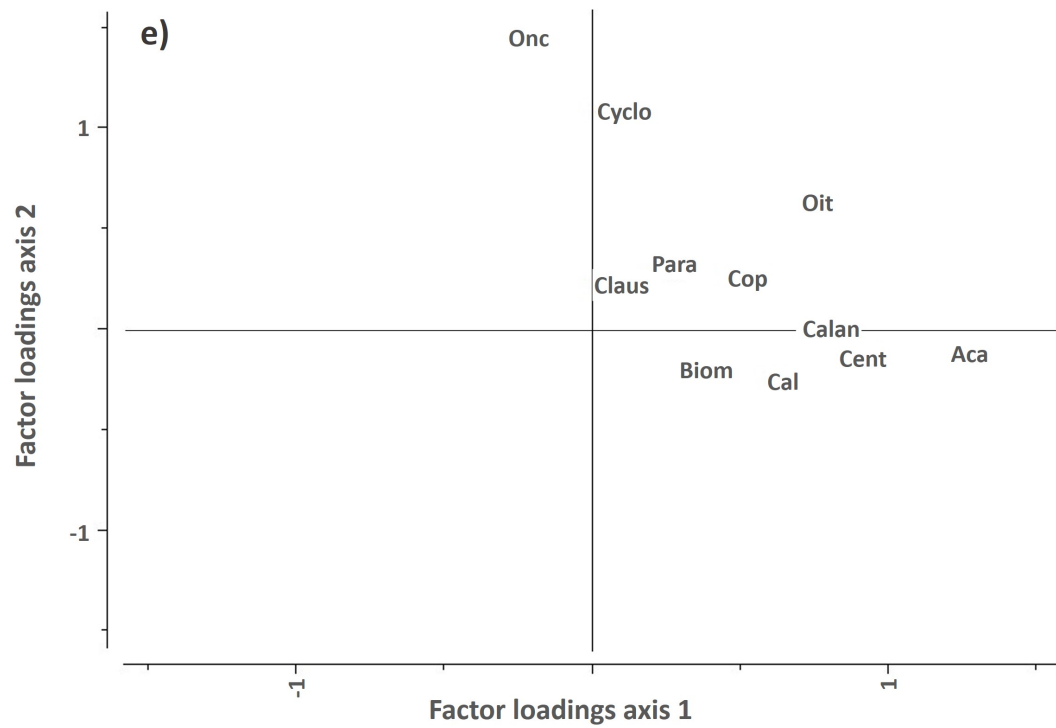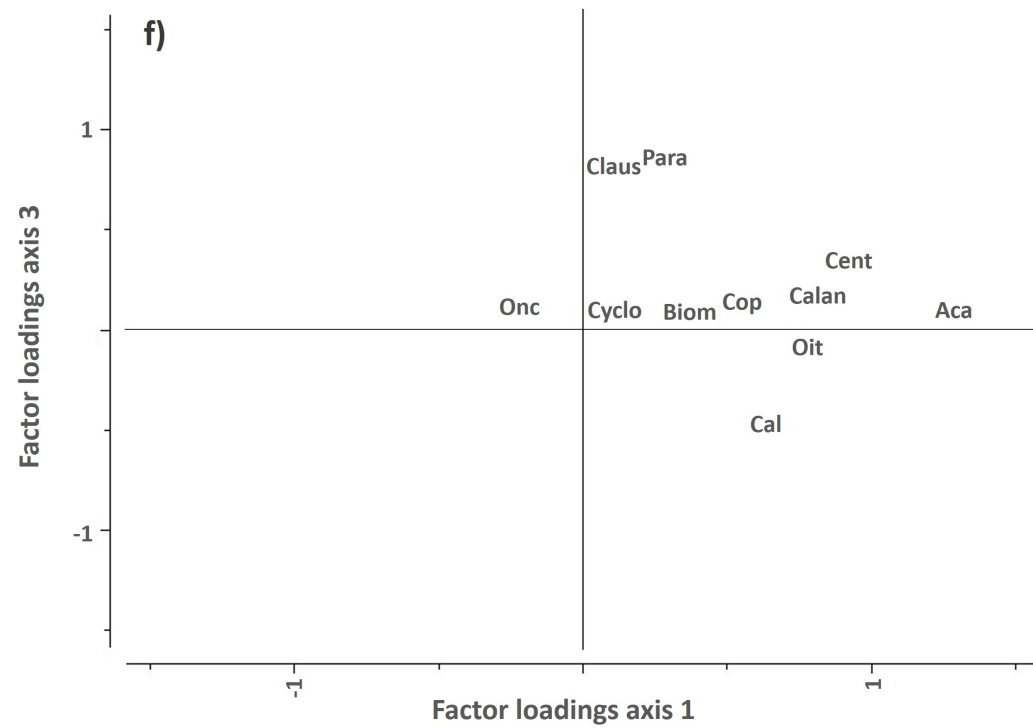

Supplement: Figure S2 — Dynamic Factor Analysis (DFA) results. Model fit and respective factor loadings (two and three common trends) applied to the most abundant taxa collected during the entire time-series for all taxa (a, c, d) and Copepoda (b, e, f), presenting the observed (black dots) and fitted (line) abundances through time in the left panels. Groups represented in the plots: zooplankton biomass (Biom), abundances of total zooplankton (TZoo), Copepoda (Cop), Mollusca (Mol), Diplostraca (Dipl), Cnidaria (Cni), Appendicularia (App), Cirripedia (Cirri), Decapoda (Dec), Chaetognatha (Chaet), Polychaeta (Poly), fish eggs/larvae (Fish), Calanoida (Calan), Cyclopoida (Cyclo), Acartia spp. (Aca), Calanus spp. (Cal), Oncaea spp. (Onc), Oithona spp. (Oit), Centropages spp. (Cent), Paracalanus spp. (Para) and Clausocalanus spp. (Claus). [file peerj-11-16387-s002.pdf]

### Contribution to fitted values

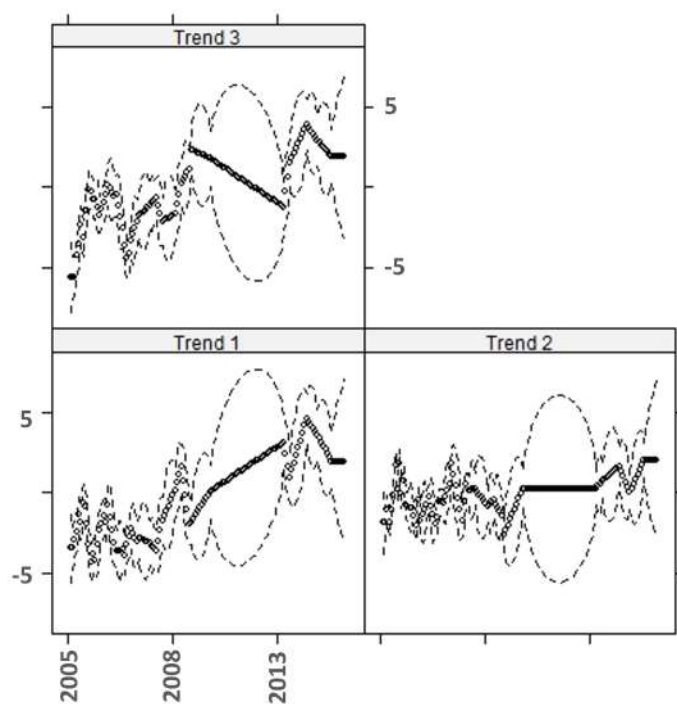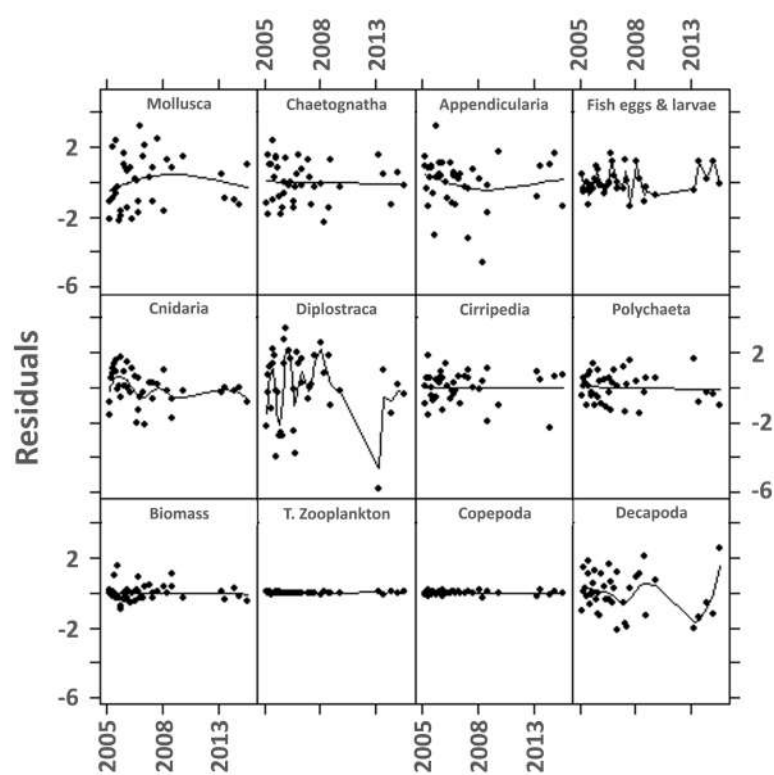

## b) Copepoda

Contribution to fitted values

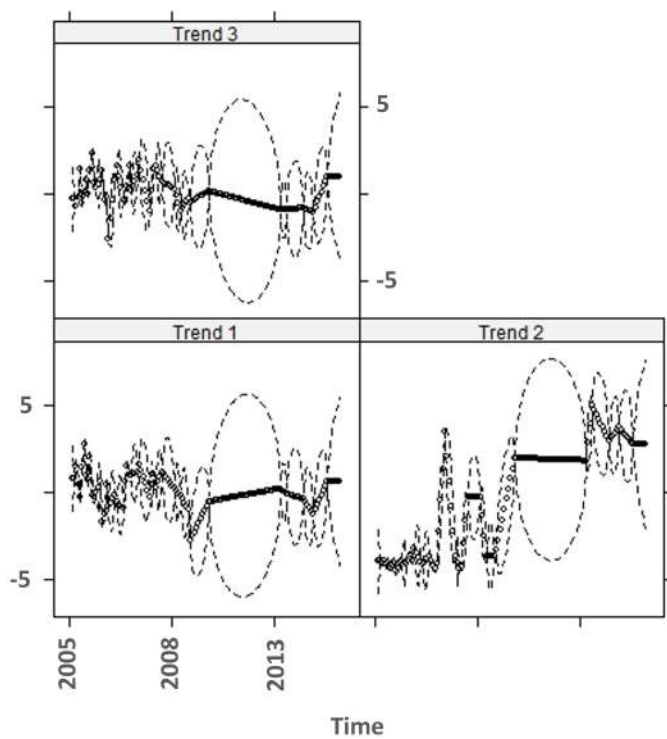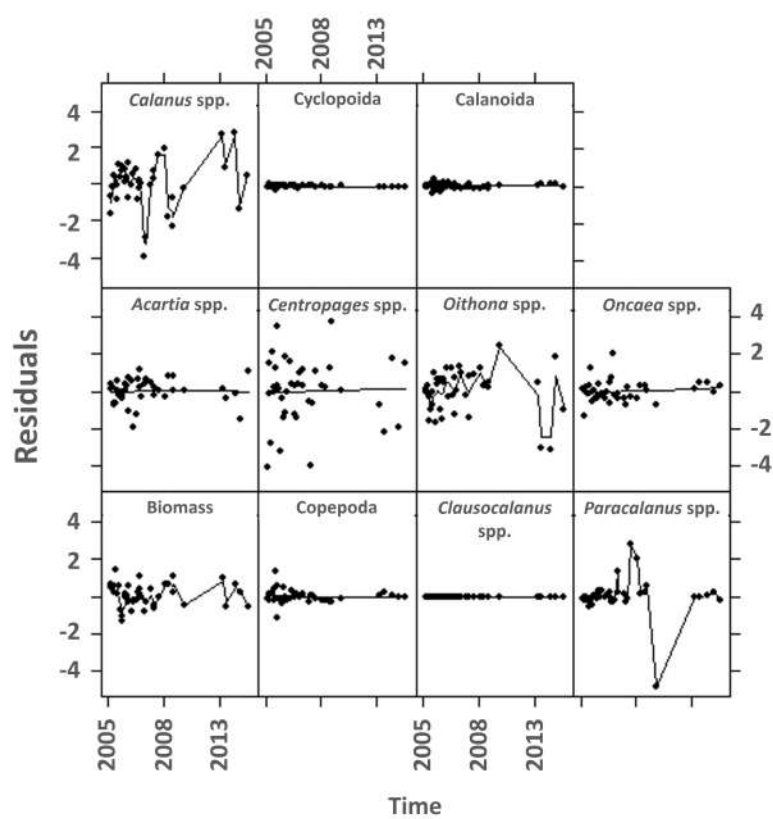

Supplement: Figure S3 — Dynamic Factor Analysis (DFA) results. Common trends and residuals (three common trends) applied to the most abundant taxa collected for the entire time-series (a) and Copepoda (b), presenting the observed (black dots) and fitted (line) abundance through time in the right panels. [file peerj-11-16387-s003.pdf]

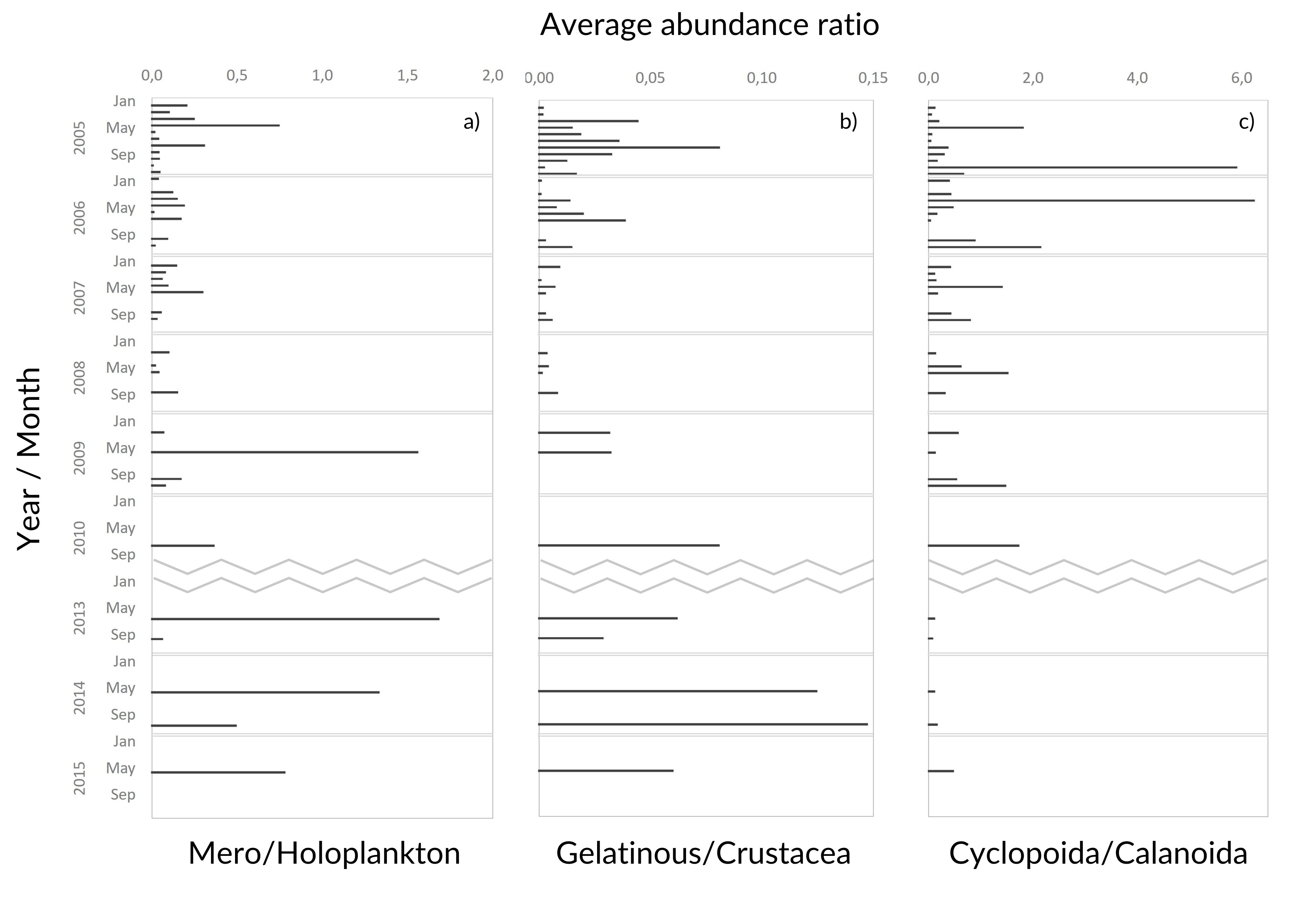

Supplement: Figure S4 — Interannual monthly variation of the abundance ratios of (a) gelatinous versus crustacean zooplankton, (b) Cyclopoida versus Calanoida copepods and (c) meroplankton versus holoplankton at the Cascais Watch site. [file peerj-11-16387-s004.jpg]

16.78 %

Component 2

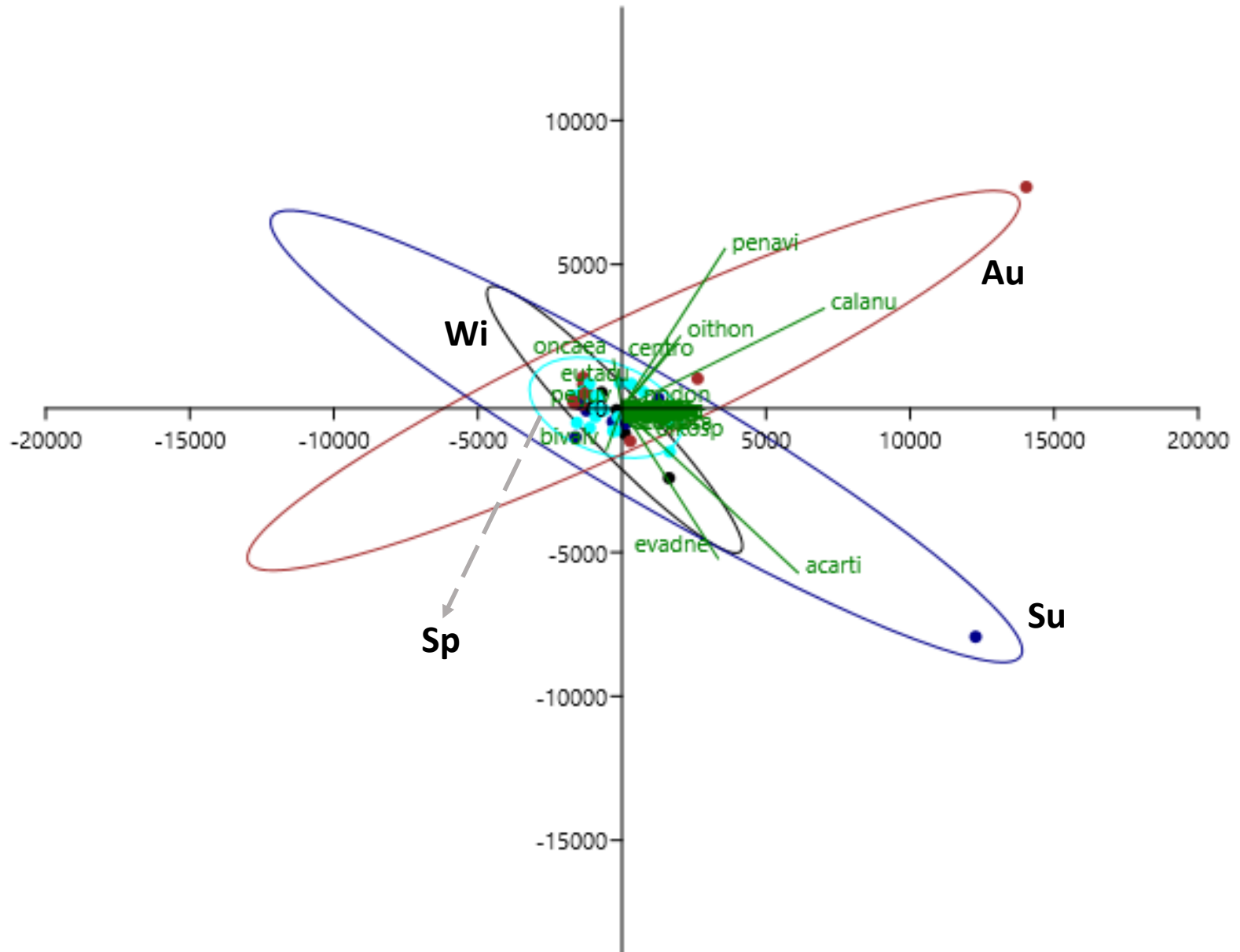

Sp

Component 1

48.02 %

Supplement: Figure S5 — Principal Component Analysis results for the first two components (64.8% of cumulative variance), representing the taxonomic groups that contributed most to the differentiation of the samples from distinct seasons, represented by ellipses –Autumn (Au), Summer (Su), Winter (Wi), Spring (Sp): Penilia avirostris (penavi), Calanus spp. (calanu), Oithona spp. (oithon), Centropages spp. (centro), Oncaea spp. (oncaea), Evadne spp. (evadne), Acartia spp. (acarti), Bivalvia (bivalv). [file peerj-11-16387-s005.pdf]
