# Supplementary material for: Zooplankton biodiversity and temporal dynamics (2005–2015) in a coastal station in western Portugal (Northeastern Atlantic Ocean)
Source: PeerJ. 2023 Nov 21;11:e16387. doi: 10.7717/peerj.16387 (PMC10668806; doi:10.7717/peerj.16387)
Supplement: Table S1 — Statistical results for the Mann-Kendall monotonic trend tests (left panel) and Lomb-Scargle periodogram (right panel) analyses regarding the most abundant zooplankton and Copepoda taxa in the CCW station, applied to the interannual monthly abundance values. Mann-Kendall Z values above 5% significance are highlighted in bold and indicate significant increasing (positive) or decreasing (negative) trends. For the Lomb-Scargle analyses, the frequencies of the power peaks are presented. [file peerj-11-16387-s006.pdf]

	Mann-Kendall				Lomb-Scargle periodogram			
	Month		Season		Month		Season	
	Z	p-value	Z	p-value	Peak frequency	p-value	Peak frequency	p-value
**Tot. Zooplankton**	-	-	0.07	0.9	0.06	0.4	0.46	0.9
**Copepoda**	-1,04	0.3	-1.02	0.3	0.06	0.4	0.08	0.96
**Decapoda**	-	**0.04**	-2.3	**0.02**	0.5	0.85	0.5	0.78
**Cnidaria**	0.9	0.4	1.2	0.2	0.1	0.97	0.04	0.7
**Diplostraca**	1.4	0.17	0.07	0.94	0.4	0.99	0.2	0.97
**Cirripedia**	-2,07	**0.04**	-2.85	**< 0.01**	0.03	0.98	0.3	0.98
**Euphausiacea**	0.89	0.37	0.92	0.36	0.5	0.7	0.5	0.4
**Polychaeta**	-2.47	**0.01**	-2.4	**0.02**	0.2	0.9	0.2	0.7
**Mollusca**	2.8	**< 0.01**	2.3	**0.02**	0.5	0.1	0.4	0.8
**Chaetognatha**	0.43	0.7	0.74	0.46	0.06	0.3	0.2	0.7
**Appendicularia**	-1.3	0.17	-0.27	0.8	0.03	0.99	0.17	0.9
**Fish larvae & eggs**	-0.9	0.4	0.4	0.7	0.03	0.3	0.05	0.8
Centropages spp.	0.7	0.5	-0.07	0.94				
Calanus spp.	-2.96	**0.003**	-2.78	**0.01**				
Clausocalanus spp.	4.2	**< 0.01**	3.5	**< 0.01**				
Paracalanus spp.	3.6	**< 0.01**	2.7	**< 0.01**				
Oithona spp.	-1,5	0.14	-1.7	0.08				
Acartia spp.	-1.7	0.09	-1.8	0.08				
Oncaea spp.	0.2	0.8	-1.1	0.3				
