# Supplementary material for: Zooplankton biodiversity and temporal dynamics (2005–2015) in a coastal station in western Portugal (Northeastern Atlantic Ocean)
Source: PeerJ. 2023 Nov 21;11:e16387. doi: 10.7717/peerj.16387 (PMC10668806; doi:10.7717/peerj.16387)
Supplement: Table S2 — Statistical results for the one-way ANOVA and Kruskal-Wallis analyses, and respective pairwise tests (Tukey and Mann-Whitney, respectively), for the main taxa detected in the CCW samples, the diversity measures and the environmental parameters (SST–Sea Surface Temperature, UI –upwelling index, Chl –chlorophyll a, Pp–precipitation) per month (Mar–March, Apr–April, Jun–June, Jul–July, Sep–September, Oct–October, Nov–November), season (Wi –winter, Sp –spring, Su –summer, Au –autumn), year and period (upwelling/downwelling). The significant tests are marked in bold and grey. [file peerj-11-16387-s007.pdf]

		Levene test	1-way ANOVA		Tukey	Kruskal-Wallis		Mann-Whitney
		p-value	F	p-value	p-value	H	p-value	p-value
Tot. zooplankton	Month	0.01	1.2	0.4	-	-	-	-
	Season	0.05	-	-	-	7.9	0.04	< 0.05 for Au vs Su
	Year	0.2	-	-	-	16.9	0.03	< 0.05 for 2005 vs 2006, 2009 and 2006 vs 2007
	Period	0.6	-	-	-	3.3	0.07	-
Zooplankton biomass	Month	< 0.01	1.6	0.15	-	-	-	-
	Season	0.1	-	-	-	3.7	0.3	-
	Year	0.4	-	-	-	12.5	0.1	-
	Period	0.5	-	-	-	2.5	0.1	-
Copepoda	Month	0.004	0.9	0.6	-	-	-	-
	Season	0.04	2.01	0.1	-	-	-	-
	Year	0.2	-	-	-	19.6	0.01	< 0.02 for 2005 vs 2006, 2009 and 2007 vs 2006, 2009
	Period	0.9	-	-	-	1.4	0.2	-
Decapoda	Month	0.02	0.7	0.7	-	-	-	-
	Season	< 0.01	3.8	0.02	< 0.03 for Wi vs Sp, Au	-	-	-
	Year	0.1	-	-	-	16.2	0.04	< 0.04 for 2005 vs 2013, 2014
	Period	0.03	0.3	0.7	-	-	-	-
Cnidaria	Month	< 0.01	1.6	0.1	-	-	-	-
	Season	0.01	3.3	0.03	p=0.05 for Su vs Au	-	-	-
	Year	0.1	-	-	-	13.5	0.1	-
	Period	0.04	2.08	0.2	-	-	-	-
Diplostraca	Month	< 0.01	1.04	0.4	-	-	-	-
	Season	< 0.01	2.2	0.1	-	-	-	-
	Year	0.05	0.4	0.9	-	-	-	-
	Period	0.4	-	-	-	0.27	0.6	-
Cirripedia	Month	< 0.01	1.38	0.2	-	-	-	-
	Season	0.1	-	-	-	8.17	0.04	< 0.01 for Au vs Sp
	Year	0.02	1	0.5	-	-	-	-
	Period	0.04	2.8	0.1	-	-	-	-
Euphausiacea	Month	< 0.01	0.6	0.8	-	-	-	-
	Season	0.02	1.5	0.2	-	-	-	-
	Year	< 0.01	5.04	< 0.01	< 0.04 for 2015 vs 2005-2010, 2014	-	-	-
	Period	0.01	2.8	0.1	-	-	-	-
Polychaeta	Month	0.07	-	-	-	16.5	0.1	-
	Season	0.01	2.5	0.07	-	-	-	-
	Year	< 0.01	1.7	0.1	-	-	-	-
	Period	0.07	-	-	-	3.4	0.06	-
Mollusca	Month	< 0.01	1.2	0,3	-	-	-	-
	Season	0.03	1.2	0.3	-	-	-	-
	Year	< 0.01	6.4	< 0.01	< 0.03 for 2013 vs 2005-2007; 2015 vs 2005-2009	-	-	-
	Period	0.01	2.5	0.1	-	-	-	-
Chaetognatha	Month	< 0.01	1.3	0.3	-	-	-	-
	Season	0.002	3.1	0.04	No significant comparisons	-	-	-
	Year	0.04	1.3	0.3	-	-	-	-
	Period	0.6	-	-	-	0.05	0.8	-
Appendicularia	Month	0.01	0.7	0.7	-	-	-	-
	Season	0.01	2.6	0.07	-	-	-	-
	Year	0.02	1.3	0.3	-	-	-	-
	Period	0.6	-	-	-	1.1	0.3	-
Fish eggs & larvae	Month	0.01	0.5	0.9	-	-	-	-
	Season	0.4	-	-	-	10.8	0.01	< 0.03 for Sp vs Su, Au
	Year	< 0.01	25.3	< 0.01	< 0.01 for 2013 vs 2005-2015; 2014 vs 2005-2013	-	-	-
	Period	0.5	-	-	-	4.3	0.04	< 0.04 Upwelling vs Downwelling

		Levene test	1-way ANOVA		Tukey	Kruskal-Wallis		Mann-Whitney
		p-value	F	p-value	p-value	H	p-value	p-value
Clausocalanus spp.	**Month**	**< 0.01**	1.03	0.4	-	-	-	-
	**Season**	**< 0.01**	2.1	0.1	-	-	-	-
	**Year**	**< 0.01**	3.4	**0.01**	**< 0.03** for 2013 vs 2005-2009	-	-	-
	Period	**< 0.01**	3.8	0.06	-	-	-	-
Paracalanus spp.	**Month**	**< 0.01**	1.3	0.3	-	-	-	-
	**Season**	**< 0.01**	1.8	0.2	-	-	-	-
	**Year**	**< 0.01**	1.6	0.2	-	-	-	-
	Period	**< 0.01**	2.5	0.1	-	-	-	-
Acartia spp.	**Month**	**< 0.01**	0.8	0.7	-	-	-	-
	**Season**	0.2	-	-	-	4.7	0.2	-
	**Year**	0.2	-	-	-	17.2	**0.03**	**< 0.02** for 2006 vs 2005-2008
	Period	0.8	-	-	-	2.4	0.1	-
Calanus spp.	**Month**	**0.002**	0.5	0.92	-	-	-	-
	**Season**	0.1	-	-	-	3.2	0.4	-
	**Year**	**0.01**	0.95	0.5	-	-	-	-
	Period	0.7	-	-	-	2.5	0.1	-
Centropages spp.	**Month**	**< 0.01**	1.2	0.3	-	-	-	-
	**Season**	**0.04**	0.9	0.5	-	-	-	-
	**Year**	0.07	-	-	-	6.8	0.6	-
	Period	0.3	-	-	-	0.2	0.7	-
Oithona spp.	**Month**	**< 0.01**	1.1	0.4	-	-	-	-
	**Season**	**0.03**	0.7	0.6	-	-	-	-
	**Year**	0.2	-	-	-	11.6	0.2	-
	Period	**0.05**	1.03	0.3	-	-	-	-
Oncaea spp.	**Month**	**0.02**	0.6	0.8	-	-	-	-
	**Season**	**< 0.01**	2.04	0.1	-	-	-	-
	**Year**	0.3	-	-	-	5.7	0.7	-
	Period	**0.004**	1.8	0.2	-	-	-	-
**Calanoida**	**Month**	**< 0.01**	0.9	0.6	-	-	-	-
	**Season**	0.1	-	-	-	3.9	0.3	-
	**Year**	0.1	-	-	-	14.2	0.08	-
	Period	0.4	-	-	-	1.4	0.2	-
**Cyclopoida**	**Month**	**0.03**	0.7	0.7	-	-	-	-
	**Season**	**< 0.01**	2.03	0.1	-	-	-	-
	**Year**	0.2	-	-	-	11.7	0.2	-
	Period	**0.003**	2.4	0.1	-	-	-	-

		Levene test	1-way ANOVA		Tukey	Kruskal-Wallis		Mann-Whitney
		p-value	F	p-value	p-value	H	p-value	p-value
**Shannon-Wiener diversity**	**Month**	0.3	-	-	-	9.6	0.6	-
	**Season**	0.3	-	-	-	4.6	0.2	-
	**Year**	0.3	-	-	-	6.6	0.6	-
	Period	0.98	-	-	-	0.09	0.8	-
**Simpson diversity**	**Month**	0.2	-	-	-	12.4	0.3	-
	**Season**	0.2	-	-	-	4.98	0.2	-
	**Year**	0.6	-	-	-	7.4	0.5	-
	Period	0.99	-	-	-	0.08	0.8	-
**Pielou's Eveness**	**Month**	0.3	-	-	-	8.8	0.6	-
	**Season**	0.5	-	-	-	2.9	0.4	-
	**Year**	0.4	-	-	-	5.8	0.7	-
	Period	0.9	-	-	-	< 0.01	0.99	-
**Menhinick taxonomic richness index**	**Month**	**0.004**	0.7	0.69	-	-	-	-
	**Season**	0.1	-	-	-	2.5	0.5	-
	**Year**	**< 0.01**	1.7	0.13	-	-	-	-
	Period	**0.02**	3.4	0.07	-	-	-	-
**Margalef's species richness**	**Month**	0.4	-	-	-	8.7	0.7	-
	**Season**	**0.04**	1.6	0.2	-	-	-	-
	**Year**	0.4	-	-	-	12	0.2	-
	Period	0.2	-	-	-	0.6	0.4	-
**SST**	**Month**	0.1	-	-	-	31.5	**< 0.01**	**< 0.04** for Mar vs Jun, Jul, Sep-Nov and Apr/May vs Jun, Oct
	**Season**	0.1	-	-	-	20.9	**< 0.01**	**< 0.04** between all seasons except Au vs Su
	**Year**	**0.02**	1.2	0.3	-	-	-	-
	Period	0.5	-	-	-	0.001	0.97	-
**UI**	**Month**	**0.003**	5.1	**< 0.01**	**< 0.02** for Oct vs Mar-Sep	-	-	-
	**Season**	0.3	-	-	-	25.2	**< 0.01**	**< 0.01** between all seasons
	**Year**	0.3	-	-	-	7.1	0.5	-
	Period	0.1	-	-	-	25.7	**< 0.01**	**< 0.01** between both periods
**Chl**	**Month**	0.07	-	-	-	4.5	0.95	-
	**Season**	0.7	-	-	-	1.8	0.6	-
	**Year**	0.6	-	-	-	15.1	0.06	-
	Period	0.9	-	-	-	< 0.01	0.99	-
**Pp**	**Month**	**< 0.01**	2.7	**0.02**	**< 0.04** for Nov vs Jun, Jul	-	-	-
	**Season**	**< 0.01**	8.9	**< 0.01**	**< 0.01** for Au vs Sp, Su	-	-	-
	**Year**	0.2	-	-	-	9.9	0.3	-
	Period	**< 0.01**	26.6	**< 0.01**	**< 0.01**	-	-	-
