# Supplementary material for: Zooplankton biodiversity and temporal dynamics (2005–2015) in a coastal station in western Portugal (Northeastern Atlantic Ocean)
Source: PeerJ. 2023 Nov 21;11:e16387. doi: 10.7717/peerj.16387 (PMC10668806; doi:10.7717/peerj.16387)
Supplement: Table S3 — Statistical results for the Principal Component Analysis, presenting the main components that explain sample variability relatively to environmental factors and taxa abundance. [file peerj-11-16387-s008.pdf]

Environmental parameters				
	Eigenvalue	% Total variance		
PC1	1.6	40.02		
PC2	1.09	27.3		
PC3	0.88	21.9		
PC4	0.4	10.8		
Eigenvectors	Factor 1	Factor 2	Factor 3	Factor 4
SST	-0.12	-0.8	0.64	0.13
Chl	-0,3	-0.6	-0.7	-0.2
UI	0.66	-0.2	-0.27	0.68
Pp	-0.68	0.2	-0.06	0.7

Month / Year		
	Eigenvalue	% Total variance
PC1	1.1	54.8
PC2	0.9	45.2

Season		
	Eigenvalue	% variance
PC1	8.6	48.02
PC2	3	16.8
PC3	2.4	13.2
PC4	1.9	10.7
