# Supplementary material for: Zooplankton biodiversity and temporal dynamics (2005–2015) in a coastal station in western Portugal (Northeastern Atlantic Ocean)
Source: PeerJ. 2023 Nov 21;11:e16387. doi: 10.7717/peerj.16387 (PMC10668806; doi:10.7717/peerj.16387)
Supplement: Table S4 — Seasonal values of frequency of occurrence (F) and mean abundance (Ab; ind m−3 ± SD) for the different taxa identified in the CCW for the entire period of 2005 to 2015. [file peerj-11-16387-s009.pdf]

Table SI Seasonal values of frequency of occurrence (F) and mean abundance (Ab; ind m^{-3} ± SD) for the different taxa identified in the entire period of 2005 to 2015.

Taxa	Winter		Spring		Summer		Autumn	
	F (%)	Ab ± SD	F (%)	Ab ± SD	F (%)	Ab ± SD	F (%)	Ab ± SD
RADIOZOA	-	-	-	-	100	0.01 ± 0.03	-	-
FORAMINIFERA n. id.	-	-	46.4	4.2 ± 14	5.9	1.1 ± 3.6	47.7	7.7 ± 18.2
Globigerina spp.	-	-	-	-	100	1.8 ± 5.7	-	-
CNIDARIA								
Hydromedusae n. id.	0.9	2.3 ± 4.1	16	11.9 ± 24.4	69.1	108.3 ± 272.2	14.1	18.4 ± 51
Siphonophorae n. id.	0.6	2.6 ± 3.1	37.1	44.2 ± 82.1	38.7	96.8 ± 91.9	23.5	48.9 ± 82.5
CTENOPHORA	-	-	11.3	0.2 ± 0.9	88.7	3.1 ± 9.7	-	-
DIPLOSTRACA (Cladocera)								
Podon spp.	0.2	1.2 ± 2	37.5	65.4 ± 94.3	38.9	142.4 ± 200.7	23.5	71.8 ± 207.6
Evadne spp	0.2	4.1 ± 6.2	20	150.8 ± 283.4	69.2	1092.5 ± 2892.8	10.6	140 ± 247.2
Penilia spp.	-	-	5.6	44.1 ± 179.8	5.8	95.6 ± 160	88.7	1226.7 ± 2692
CIRRIPEDIA								
Cirripedia (nauplius)	16.8	268.5 ± 285.3	58.5	267.4 ± 448.8	16.5	158.1 ± 194.7	8.3	66.4 ± 65.8
Cirripedia (cyprids)	6.7	19 ± 28.2	75.2	60.8 ± 97.2	17.2	29.2 ± 56.4	0.9	1.3 ± 3.3
COPEPODA								
Copepoda (egg)	-	-	46.6	1.8 ± 8.2	-	-	53.4	3.6 ± 8.7
Copepoda (nauplius)	10.6	158.7 ± 217.3	74.4	316.8 ± 670	10	89.3 ± 78.5	5	37.1 ± 77.5
Calanoida (copepodite)	-	-	11	35.8 ± 164.3	46.8	318.9 ± 514.6	42.2	239.8 ± 530.7
Calanus spp.	8.2	657.3 ± 315.1	33.4	761.1 ± 832.1	26.9	1285.3 ± 2152	31.5	1256.5 ± 3571.9
Calanus helgolandicus	1.6	13.6 ± 25.3	79.2	190.9 ± 348.1	16.2	82 ± 175.5	2.9	12.4 ± 22.6
Calanoides carinatus	-	-	86.5	20.8 ± 79.2	13.5	6.8 ± 21.5	-	-
Mesocalanus tenuicornis	-	-	27.3	3.9 ± 12.3	69.6	21 ± 64.4	3.2	0.8 ± 2.8
Neocalanus gracilis	-	-	-	-	100	15.2 ± 44.6	-	-
Calocalanus spp.	8.4	3.7 ± 9.1	10.9	1.4 ± 3.6	48	12.7 ± 22.5	32.8	7.2 ± 21.6
Clausocalanus spp.	-	-	16.6	29.8 ± 91.6	9.4	35.3 ± 57.1	74	231.9 ± 465.2
Paracalanus spp.	-	-	12.6	38.2 ± 98.7	11.3	72.1 ± 194	76.1	404.3 ± 666.7
Eucalanus spp.	14.1	6 ± 5.5	44	5.3 ± 11	14.5	3.7 ± 10.5	27.4	5.8 ± 19.4
Anomalocera patersoni	-	-	-	-	100	3.4 ± 10.8	-	-
Metridia lucens	-	-	-	-	62.2	1.1 ± 3.3	37.8	0.5 ± 1.9
Pleuromamma spp.	15.6	0.7 ± 1.7	20.9	0.3 ± 1.2	51.8	1.4 ± 4.5	11.8	0.3 ± 0.9
Diaixis spp.	-	-	-	-	-	-	100	0.4 ± 1.3
Acartia spp.	14.8	1692.7 ± 1903.6	31.3	1020.7 ± 1021.1	32.80	2245.2 ± 3264.7	21.1	1200.4 ± 1847.9
Euchaeta spp.	-	-	-	-	100	1.2 ± 2.5	-	-
Candacia spp.	2	0.7 ± 1.7	41.6	4.1 ± 9.4	32	6.7 ± 14.1	24.4	4.2 ± 10.7
Pseudocalanus elongatus	-	-	-	-	-	-	100	0.8 ± 2.6
Pseudocalanus spp.	-	-	-	-	100	0.6 ± 1.8	-	-
Centropages spp.	1.5	32.9 ± 44.1	22.7	143.7 ± 321.5	26	345 ± 395.8	49.8	551.2 ± 1275.4
Isia clavipes	-	-	42.6	1.5 ± 5	57.4	4.2 ± 9	-	-
Temora longicornis	2.1	31.5 ± 61.5	63.8	272.4 ± 463.6	28.7	257.7 ± 332.4	5.4	40.4 ± 87.4
Temora stylifera	0.1	0.4 ± 0.9	9	7.7 ± 21.9	7.8	14.2 ± 38.2	83.1	125.5 ± 160.3
Oithona spp.	8.8	295.8 ± 259.7	28.6	274 ± 387.9	20.7	416 ± 433.6	42	703 ± 1348.5
Harpacticoida n. id.	5.6	53.3 ± 45.4	66.5	180.8 ± 279.9	9.9	56.7 ± 42	18	85.7 ± 130.7
Aegisthus mucronatus	-	-	100	0.3 ± 1.5	-	-	-	-
Clytemenestra spp.	39.6	1.1 ± 2.7	33.6	0.3 ± 1.2	-	-	26.8	0.4 ± 1.3
Microsetella spp.	-	-	34.3	0.8 ± 2.3	33.2	1.7 ± 5.3	32.5	1.4 ± 4.3
Euterpina acutifrons	5.6	52.2 ± 45.8	67.2	179.4 ± 280.2	9.4	52.8 ± 40.8	17.8	83.2 ± 131.4
Monstrilla spp.	-	-	-	-	100	0.8 ± 1.7	-	-
Corycaeus spp.	21.8	62.5 ± 55.3	49	40.1 ± 45.6	12.5	21.5 ± 20	16.7	24 ± 26.3
Oncaea spp.	2.3	156.5 ± 169.9	54.8	1084.1 ± 1390	7.3	304.2 ± 309.5	35.6	1233.4 ± 1837.9
Copepoda (parasite)	-	-	-	-	-	-	100	0.5 ± 1.9
OSTRACODA	41.5	0.9 ± 0.8	-	-	27.8	0.4 ± 1.1	30.7	0.3 ± 0.7
STOMATOPODA	-	-	100	0.006 ± 0.03	-	-	-	-
Platysquilla eusebia	-	-	94.2	0.02 ± 0.1	5.8	0.002 ± 0.007	-	-
MYSIDA	-	-	-	-	37.2	1.2 ± 2.1	62.8	1.7 ± 3.1
AMPHIPODA	-	-	-	-	-	-	100	0.1 ± 0.2
ISOPODA	-	-	-	-	-	-	100	0.1 ± 0.2
CUMACEA	-	-	75.7	0.1 ± 0.5	-	-	24.3	0.1 ± 0.2
EUPHAUSIACEA								
Euphausiacea (nauplius)	-	-	40.2	2.1 ± 9.8	57.6	6.4 ± 19.7	2.3	0.2 ± 0.7
Euphausiacea (caliptopis)	6.4	7 ± 6.9	63.9	19.9 ± 32.1	21.5	14.1 ± 21.6	8.2	4.5 ± 7
Euphausiacea (furcilia)	3.7	1.1 ± 2	26.3	2.2 ± 4.9	49.4	8.6 ± 14	20.6	3 ± 9.6
DECAPODA								
Dendrobranchiata	28.3	0.2 ± 0.4	46.8	0.1 ± 0.3	23.9	0.1 ± 0.1	1	-
Caridea	19.2	10.8 ± 13.4	32.4	5.2 ± 5.5	35.5	12 ± 16.8	12.9	3.6 ± 4
Gebiidea and Axiidea	5.3	0.1 ± 0.2	71.7	0.3 ± 0.5	7.6	0.1 ± 0.1	15.4	0.1 ± 0.2
Achelata and Polychelida	9.5	0.02 ± 0.1	63	0.04 ± 0.1	9.3	0.01 ± 0.04	18.2	0.02 ± 0.03
Anomura	12	13.4 ± 22.4	45.4	14.5 ± 15.3	26.8	18 ± 32.9	15.7	8.8 ± 12.3
Brachyura	59.5	154 ± 149.1	23.4	17.3 ± 18.4	11.2	17.3 ± 21.7	6	7.7 ± 11
POLYCHAETA	13.4	16.04 ± 15.9	45.6	15.6 ± 16.2	29.1	21 ± 27.5	11.9	7.1 ± 11.4
MOLLUSCA								
Bivalvia (larvae)	0.04	2.1 ± 4.5	53.4	748.4 ± 1730.5	34.3	1008.1 ± 2105.9	12.2	300.1 ± 550.5
Gastropoda	-	-	38.3	14.1 ± 41.1	10.3	7.9 ± 25.1	51.5	33.1 ± 80.5
Pteropoda	12	2.7 ± 4	53.6	3.45 ± 7.2	5	0.7 ± 1.8	29.5	3.3 ± 8.3
Limacina spp.	-	-	-	-	100	2.1 ± 6.7	-	-
CHAETOGNATHA	1.3	2.6 ± 4.6	21.9	12.9 ± 14.9	42.8	52.8 ± 55.6	34	34.9 ± 43.1
ECHINODERMATA								
Ophiurida	-	-	43.6	0.4 ± 1.5	-	-	56.4	0.9 ± 3.2
Echinodermata (larvae)	-	-	31.5	3.2 ± 11.4	24.2	5.1 ± 16.1	44.3	7.9 ± 18.1
PHRONIDA	-	-	-	-	100	0.01 ± 0.03	-	-
THALIACEA								
Salpida	1.1	1 ± 2.4	23.4	5.6 ± 25.7	75.5	38.3 ± 63.7	-	-
Doliolida	0.9	1.4 ± 3.5	43.8	18.6 ± 74.5	0.7	0.6 ± 2	54.5	40.5 ± 61.3
APPENDICULARIA								
Oikopleura spp.	21	418.2 ± 732.5	28.8	163.5 ± 194.6	28.3	337.8 ± 480.4	21.9	217.4 ± 287.3
Fritillaria spp.	11	16.6 ± 24.2	30.8	13.2 ± 22.7	52.9	47.7 ± 51.1	5.2	3.9 ± 7.5
Fish eggs	4.6	3.5 ± 3	72.2	15.3 ± 22.9	7.7	3.4 ± 3.6	15.5	5.8 ± 14.6
Fish larvae	7.5	0.9 ± 1	34.7	1.2 ± 2.8	24.3	1.8 ± 3.4	33.5	2.1 ± 5.1
Fish juveniles	100	0.002 ± 0.005	-	-	-	-	-	-
